# Erect wing regulates synaptic growth in *Drosophila *by integration of multiple signaling pathways

**DOI:** 10.1186/gb-2008-9-4-r73

**Published:** 2008-04-17

**Authors:** Irmgard U Haussmann, Kalpana White, Matthias Soller

**Affiliations:** 1 School of Biosciences, University of Birmingham, Edgbaston, Birmingham, B15 2TT, UK; 2 Department of Biology and Volen Center for Complex Systems, Brandeis University, Waltham, MA 02454, USA

## Abstract

**Background:**

Formation of synaptic connections is a dynamic and highly regulated process. Little is known about the gene networks that regulate synaptic growth and how they balance stimulatory and restrictive signals.

**Results:**

Here we show that the neuronally expressed transcription factor gene *erect wing* (*ewg*) is a major target of the RNA binding protein ELAV and that EWG restricts synaptic growth at neuromuscular junctions. Using a functional genomics approach we demonstrate that EWG acts primarily through increasing mRNA levels of genes involved in transcriptional and post-transcriptional regulation of gene expression, while genes at the end of the regulatory expression hierarchy (effector genes) represent only a minor portion, indicating an extensive regulatory network. Among EWG-regulated genes are components of Wingless and Notch signaling pathways. In a clonal analysis we demonstrate that EWG genetically interacts with Wingless and Notch, and also with TGF-β and AP-1 pathways in the regulation of synaptic growth.

**Conclusion:**

Our results show that EWG restricts synaptic growth by integrating multiple cellular signaling pathways into an extensive regulatory gene expression network.

## Background

Synaptic connections are formed during development and continue to be remodeled in the adult nervous system. Such morphological changes are implicated as the cellular basis of neuronal information processing and storage in the brain [1]. Although numerous molecules that affect synaptic growth have been identified, little is known about how expression of the genes encoding these is orchestrated by cellular signaling to regulate this form of synaptic plasticity.

Several signaling pathways with prominent roles in development have also been shown to regulate synaptic growth. These include Wnt/Wingless and transforming growth factor (TGF)-β/bone morphogenetic protein (BMP) signaling pathways (reviewed in [2,3]), as well as the *jun *kinase pathway [4]. All of these pathways stimulate synaptic growth at neuromuscular junctions (NMJs) in *Drosophila *larvae [5-9], a model system for synaptic plasticity of glutamergic synapses [10]. In addition, Notch (N) signaling has recently also been implicated in plasticity due to impaired memory formation [11-13]. A major focus of these studies has been the identification of the transcription factors regulated by these signaling cascades. Prominent roles have been attributed to immediate early genes such as the transcription factors *fos *and *jun *[4,14-16], as well as the SMAD and co-SMAD homologues *mad *and *medea *in *Drosophila *[5-9]. Although much has been learned about how extracellular signals are transduced to the nucleus and regulate transcription factors, relatively little is known about gene networks and their organization, and how they operate in response to cellular signaling to mediate synaptic growth.

To delineate the nuclear response underlying presynaptic regulation of synaptic growth in a *Drosophila *model, we focused on the role of the transcription factor Erect wing (EWG), a homologoue of human NRF-1 [17,18]. A salient feature in the regulation of *ewg *expression is the elaborate control by ELAV, a post-transcriptional regulator expressed in neurons of *Drosophila *that is required for EWG protein expression [19-22]. The human homologue of ELAV, HuD, has previously been implicated in the regulation of synaptic plasticity [20,23], but the molecular and cellular consequences of increased expression of HuD are largely unknown. Consistent with a potential role in presynaptically regulating synaptic growth, EWG protein is expressed in all neurons, and transiently also in indirect flight muscles [17,24,25]. *ewg *mutant embryos are unable to exhibit coordinate larval movements and fail to hatch.

Here we show that the transcription factor gene *ewg *is a major target of the RNA binding protein ELAV and that EWG restricts synaptic growth at NMJs. This novel pathway primarily acts through EWG-up-regulated genes involved in either transcriptional or post-transcriptional regulation of gene expression. Analysis of synaptic growth in mutants of genes differentially regulated in *ewg*^*l1*^mutants shows that these genes are involved in both stimulatory and restrictive pathways. We further show that *ewg *genetically interacts with multiple signaling pathways in synaptic growth regulation in *Drosophila*. Our data suggest, therefore, that multiple cellular signaling pathways are connected with EWG regulation of synaptic growth in an extensive regulatory gene network.

## Results

### Erect wing restricts synaptic growth at neuromuscular junctions

**Figure 1:**
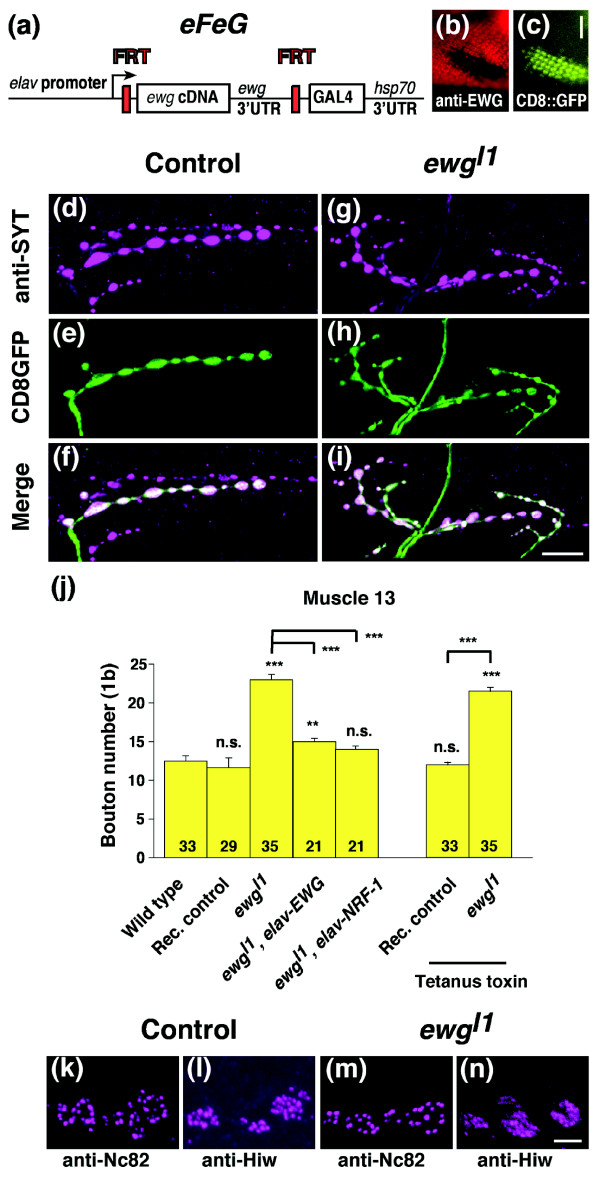
Erect wing restricts synaptic growth at third instar neuromuscular junctions. **(a) **Schematic of the *eFeG *construct used for clonal analysis of *ewg*^*l1*^, an embryonic lethal allele. **(b,c) **FLP/FRT mediated recombination in photoreceptor neurons in third instar larval eye disc. Note that CD8::GFP is expressed (c) in the *ewg*^*l1*^clone (b). The scale bar in (c) is 25 μm. **(d-i) **NMJs of control and *ewg*^*l1*^clones in third instar larvae. Clones of controls (d-f, in *ewg*^*l1*^*eFeG/+; hs-flp/+ UAS-CD8::GFP/+ *females, rec. control in (j)) and of *ewg*^*l1*^(g-i, in *ewg*^*l1*^*eFeG/Y; hs-flp/+ UAS-CD8::GFP/+ *males) were stained with anti-SYT or with anti-CD8 antibodies to visualize synaptic growth defects of type 1b boutons at muscle 13. The scale bar in (i) is 20 μm. **(j) **Quantification of synaptic growth defects in *ewg*^*l1*^mutant neurons. Shown are means of bouton numbers (type 1b at muscle 13) with standard errors (n = 21-35). Rec. control refers to clones made in the presence of one copy of *ewg*^+ ^as in *ewg*^*l1*^*eFeG/+; hs-flp/+ UAS-CD8::GFP/+ *females. Tetanus toxin was expressed from a *UAS *transgene in clones by the recombined *eFeG *construct. Statistical significance of differences from comparisons with wild type is shown on top of bars (***p < 0.0001, **p < 0.001, n.s. for non significant). Other relevant comparisons are marked by horizontal bars with the statistical significance indicated on the side. **(k-n) **Distribution of synaptic markers is normal at *ewg*^*l1*^NMJs. Active zones were stained with anti-Nc82 at wild type (k) or *ewg*^*l1*^NMJs (m) and periactive zones were stained with anti-Highwire at wild type (l) or *ewg*^*l1*^NMJs (n). The scale bar in (n) is 1 μm.

To examine *ewg *mutants for synaptic growth defects at third instar NMJs, we used a clonal analysis strategy in mosaic animals. For this analysis we made the following rescue construct, termed *eFeG*. The *ewg *cDNA was flanked by FRT sites and fused to an *elav *promoter. To visualize recombination, the sequence of yeast GAL4 was inserted downstream of the FRT-*ewg*-FRT cassette. FLP/FRT mediated recombination will result in loss of the *ewg *cDNA and lead to expression of GAL4 in neurons that can be visualized in the presence of a *UAS-CD8::GFP *transgene (Figure 1a,c,e-i) [17,24,25]. The functionality of *eFeG *is shown in a clone induced in photoreceptor neurons in *ewg*^*l1*^, a null allele [24]. Upon loss of the *ewg *cDNA in the clone, CD8::GFP is expressed and EWG expression is lost (Figure 1b,c). For the analysis of third instar NMJs, recombination was induced in late embryogensis (14-16 h after egg laying). Larvae bearing EWG null clones move indistinguishably from their balancer carrying siblings, pupate normally and many adults hatch. These adults are, however, impaired in walking.

At third instar NMJs, EWG deficient motorneurons have an increased number of synaptic boutons that look morphologically normal as visualized with an antibody against synaptotagmin, a marker for synaptic vesicles [26] (Figure 1d-i). Quantification of type 1b boutons at muscle 13 revealed about an 85% increase in bouton number (*p *< 0.0001; Figure 1j) compared to wild-type or balancer carrying siblings containing one wild-type copy of *ewg *(rec. control in Figure 1j). Since *ewg *is located on the X chromosome and we used males, we also quantified type 1b bouton numbers at muscle 13 in *ewg*^*l1*^females transheterozygous for a hypomorphic *P *element insertion, *P{lacW}ewg*^*G*1518 ^(18.8 ± 0.45, n = 18, *p *< 0.0001 for comparison with wild type). Significant effects were also observed at muscles 4, 6/7 and 12 (29%, 30% and 45% increase compared to wild type, *p *≤ 0.001). Besides overgrowth, NMJs of EWG deficient clones appeared normal and staining with markers for active (Nc82) and periactive (Highwire) zones, for microtubules (Mab 22C10) or post-synaptic specializations (anti-DLG) did not reveal obvious differences (Figure 1k-n and data not shown). The role of EWG in synaptic growth regulation is cell-autonomous, since bouton numbers of non-recombined neurons in mosaic animals were indistinguishable from wild type (data not shown). Inhibition of synaptic transmission by expression of tetanus toxin in EWG deficient neurons did not affect overgrowth, demonstrating that overgrowth is not a result of compensatory signals from the muscle due to changes in neuronal activity (Figure 1j). Similarly, expression of tetanus toxin also did not affect synaptic growth in wild-type clones (Figure 1j).

To demonstrate that synaptic overgrowth of EWG deficient neurons is a result of *ewg *loss of function (LOF), we added a rescue construct where an *elav *promoter drives the *ewg *cDNA (*elav-EWG*) [17]. Presence of this construct significantly reduced the number of type 1b boutons at muscle 13 compared to EWG deficient neurons (*p *< 0.0001; Figure 1f), but rescue is not complete compared to wild-type neurons (*p *< 0.001; Figure 1f). Since *ewg *is strongly homologous to human NRF-1 (80% in the DNA binding domain [18]), we tested if expression of NRF-1 under an *elav *promoter can rescue the synaptic growth defect of *ewg*. *elav-NRF-1 *transgenes fully rescued synaptic growth defects of *ewg*^*l1*^mutants (*p *< 0.0001), resulting in bouton numbers that were not significantly different from wild type (Figure 1j). *elav-NRF-1 *transgenes also fully rescued embryonic lethality of *ewg*^*l1*^mutant embryos (89 ± 8%, n = 5 independent transgene insertions). *ewg*^*l1*^*elav-NRF-1 *animals develop to pharate adults, but fail to hatch.

**Figure 2:**
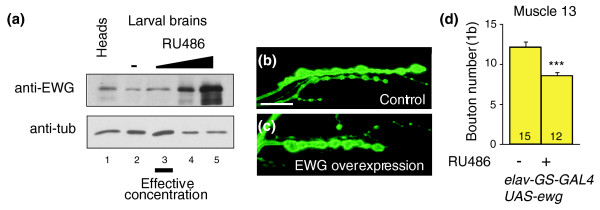
Conditional overexpression of *ewg *reduces synaptic growth. **(a) **Western blot of EWG in larval brains upon induced expression. EWG levels in larval brains were compared between *elav-GS-GAL4 UAS-ewg *animals fed with RU486 (1.2, 4.8 and 19.2 μg/10 ml food, lanes 3-5) and uninduced animals (lane 2), and to adult heads (lane 1). Note that a two-fold overexpression of EWG is effective to reduce synaptic growth (lane 3) compared to wild type (lane 2). **(b,c) **NMJs of control and EWG overexpressing animals. Shown are NMJs at muscle 13 of uninduced (b) or induced (c, 1.2 μg RU486/10 ml food) *elav-GS-GAL4 UAS-ewg *animals stained with anti-HRP antibodies. The scale bar in (b) is 20 μm. **(d) **Quantification of synaptic growth defects with excess EWG. Shown are means of bouton numbers (type 1b at muscle 13) with standard errors (n = 12-15) of uninduced (b) or induced (c, 1.2 μg RU486/10 ml food) *elav-GS-GAL4 UAS-ewg *animals. Statistically significant differences are indicated by asterisks (***p < 0.0001).

Since *ewg *LOF results in synaptic overgrowth, we wanted to know if overexpression of *ewg *results in reduced synaptic growth. To separate *ewg *function in early neuronal differentiation from its role in synaptic growth regulation and to apply physiological concentrations of EWG, we used an inducible neuron-specific GAL4 driver (*elav-GeneSwitch-GAL4*, *elav-GS-GAL4 *[27]) to express EWG from a *UAS-ewg *transgene. Concentration dependent inducibility of the *elav-GS-GAL4 *was verified by western blots (Figure 2a). At NMJs, numbers of type 1b boutons at muscle 13 were about 30% reduced compared to wild type, and at muscle 12 about 45% reduced (*p *< 0.0001; Figure 2b-d) when EWG expression was about two-fold increased compared to wild type (Figure 2a, lane 3 versus lane 2). Higher expression levels of EWG did not further reduce synaptic growth, but resulted in pupal lethality. Controls with induced *elav-GS-GAL4 *(but no *UAS-ewg*), or *UAS-ewg *alone had wild-type bouton numbers (data not shown).

### *ewg *is a major ELAV target in post-embryonic development

**Figure 3:**
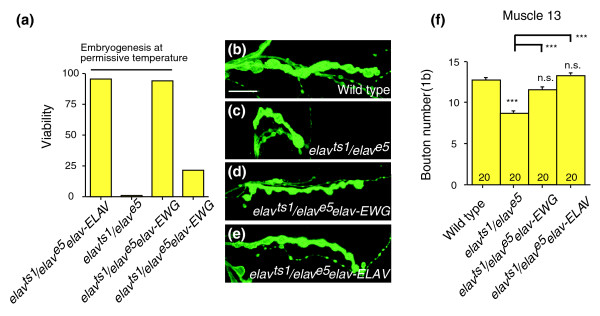
Erect wing rescues viability and synaptic growth defects of *elav *mutants. **(a) **Rescue of viability of *elav *mutants by *ewg *transgenes. An *elav-EWG *transgene fully rescues viability of a temperature sensitive *elav *allele (*elav*^*ts1*^transheterozygous for the *elav*^*e5*^, a null allele); when early functions in neuronal differentiation are provided by rearing flies at the permissive temperature (three days at 18°C and then at 25°C, n = 250-350 animals per genotype). **(b-f) **Rescue of synaptic growth defects of *elav *mutants by EWG. Synaptic growth in *elav*^*ts1*^/*elav*^*e5*^mutants (c,f) is significantly reduced (*p *≤ 0.0001) when reared at the restrictive temperature during larval life compared to wild type (b,f) and is rescued by *elav-EWG *(d,f) and *elav-ELAV *(e,f). Bouton numbers (type 1b at muscle 13) in (f) are shown as means with standard errors (n = 21-35). Statistical significance of differences from comparisons with wild type is shown on top of bars (***p < 0.0001, n.s. for non significant). Other relevant comparisons are marked by horizontal bars with the statistical significance indicated on the side. The scale bar in (b) is 20 μm.

In the presence of the RNA binding protein ELAV, the last *ewg *intron is spliced, resulting in expression of EWG protein [19,21]. Since the *ewg *gene encodes a transcription factor, EWG could potentially regulate a large portion of genes that are also regulated by ELAV, and therefore rescue *elav *mutants. To test if *elav *mutants are rescued by EWG, we used animals transheterozygous for the *elav*^*ts1*^temperature sensitive allele and for the null allele *elav*^*e5*^[28]. When early functions of ELAV in neuronal differentiation were allowed by rearing embryos at the permissive temperature, *elav-EWG *fully rescued the lethality of *elav*^*ts1*^/*elav*^*e5*^flies (Figure 3a). The rescued *elav*^*ts1*^/*elav*^*e5*^; *elav-EWG *animals, however, showed motor defects and were flightless, suggesting that the RNA binding protein ELAV regulates additional genes. Given the prominent NMJ phenotype of *ewg *mutants, we next tested if ELAV is also involved in regulating synaptic growth. At NMJs, *elav*^*ts1*^/*elav*^*e5*^animals showed a significant reduction of bouton numbers compared to wild type (*p *< 0.0001; Figure 3c,f), a phenotype opposite to the *ewg *mutant phenotype. Synaptic growth defects of *elav*^*ts1*^/*elav*^*e5*^animals, however, were fully rescued to wild-type levels by an *elav-EWG *transgene (*p *< 0.0001 for *elav*^*ts1*^/*elav*^*e5*^; *elav-EWG *compared with *elav*^*ts1*^/*elav*^*e5*^and not significantly different from wild type; Figure 3d,f). As expected, an *elav-ELAV *transgene also fully rescued synaptic growth defects of *elav*^*ts1*^/*elav*^*e5*^animals (*p *< 0.0001 for *elav*^*ts1*^/*elav*^*e5*^; *elav-ELAV *compared with *elav*^*ts1*^/*elav*^*e5*^and not significantly different from wild type; Figure 3d,f). These results indicate overlapping stimulatory and restrictive pathways in regulating synaptic growth that are integrated through the transcriptional regulator EWG (see below). Boutons in *elav*^*ts1*^/*elav*^*e5*^animals appeared normal and staining with markers for active (Nc82) and periactive (Highwire) zones, for microtubules (Mab 22C10) or post-synaptic specializations (anti-DLG) did not reveal obvious differences (data not shown).

### Identification of genes differentially regulated in *ewg*^*l1*^mutants

**Figure 4:**
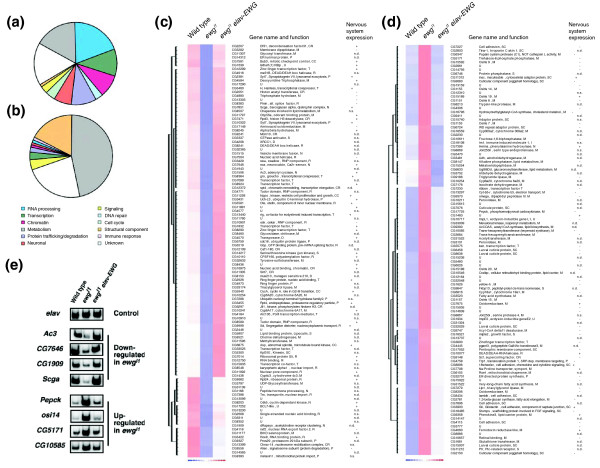
Genes differentially regulated in late embryos of *ewg*^*l1*^mutants. **(a,b) **Functional classification of genes differentially regulated in late embryos of *ewg*^*l1*^mutants. Down-regulated (a) and up-regulated (b) genes were classified according to Gene Ontology processes. **(c,d) **List of genes differentially regulated in late embryos of *ewg*^*l1*^mutants, giving functional classification and nervous system expression. Hierarchical clustering of normalized expression levels of down-regulated (c) and up-regulated (d) genes in *ewg*^*l1*^embryos compared with wild type and *ewg*^*l1*^embryos rescued with *elav-EWG*. Gene names and functional categories are shown to the right together with nervous system expression data determined by RNA *in situ *hybridization (+, expressed in the nervous system; -, not expressed in the nervous system; n.s., no staining; n.d., not determined). Differential expression is visualized by blue (down-regulation) and red (up-regulation). **(e) **Quantitative RT-PCR of selected genes differentially regulated in *ewg*^*l1*^mutants. PCR products using ^32^P labeled forward primers from genes up-regulated in *ewg*^*l1*^mutants *Ac3*, *CG7646*, *CG1909 *and *Srca *(cycles 28, 24, 28 and 28), and from genes down-regulated in *ewg*^*l1*^mutants *Pepck*, *osi14*, *CG5171 *and *CG10585 *(cycles 26) were analyzed on 6% polyacrylamide gels. *elav*: control (cycle 28).

To assess how the transcription factor EWG regulates synaptic growth, we identified genes differentially regulated in *ewg*^*l1*^mutants using cDNA microarrays. We hand-selected *ewg*^*l1*^late stage embryos that differ from their siblings by the lack of green fluorescent protein (GFP), extracted and amplified polyA RNA, and hybridized cDNA microarrays. To exclude genes differentially regulated due to genetic background and to validate genes differentially regulated in *ewg*^*l1*^mutants at a genomic scale, we included *ewg*^*l1*^animals rescued by *elav-EWG*. From these experiments (n = 4, except for *ewg*^*l1*^n = 5), mRNA expression levels of 107 genes were significantly down-regulated (Figure 4c) and of 107 genes were significantly up-regulated (Figure 4d) in *ewg*^*l1*^embryos (*p *≤ 0.01; changes in expression levels are shown in Table S3 of Additional data file 1) compared to both wild-type and *ewg*^*l1*^*elav-EWG *embryos. Quantitative RT-PCR analysis from selected genes that were down-regulated (*Ac3*, *CG7646*, *CG1909*, *Srca*) or up-regulated (*Pepck*, *osi14*, *CG5171*, *CG10585*) in *ewg*^*l1*^mutant embryos further confirmed the findings from the microarray expression analysis (Figure 4e), for example, expression levels were reduced or increased in *ewg*^*l1*^mutant embryos compared to the control gene *elav*, but restored to wild-type levels in the presence of the *elav-EWG *transgene.

Clustering differentially regulated genes revealed that down-regulated genes fall into several functional classes with a preference for genes involved in the regulation of gene expression (40%, 43 of 107; Figure 4a,c), while up-regulated genes are enriched for genes involved in basic cellular metabolism (47%, 50 of 107; Figure 4b,d). Surprisingly, only a minor portion of genes (4%, 8 of 214; Figure 4c,d) could be loosely defined as 'neuronal effector genes' (genes at the end of the regulatory expression hierarchy in neurons and associated with neuron specific functions) with known or potential functions in synaptic growth regulation, for example, *Ac3*, encoding an adenylyl cyclase, *CG1909*, encoding a *Drosophila *Rapsyn homologue, *CG7646*, encoding a Ca^2+ ^sensor, and the gene encoding Henna, a phenylalanine hydroxylase involved in dopamine and serotonin synthesis. Candidates for mediating the synaptic growth phenotype from the remaining functional classes include *groucho *(*gro*), encoding a transcriptional repressor of Wg and N signaling, and genes encoding a number of cell adhesion molecules (*CG7227*, *18w*, *CG8434*, *CG7896*, *Gli*, *CG4115*).

Synaptic growth defects in *ewg *mutants are cell-autonomous, suggesting that differentially expressed genes involved in regulating synaptic growth are also expressed in the nervous system. We therefore analyzed RNA expression patterns in late stage embryos from a representative number of genes differentially regulated in *ewg*^*l1*^mutants (66%, 74 down- and 68 up-regulated genes; RNA *in situ *expression patterns are mostly available from the Berkeley *Drosophila *Genome Project (BDGP)). RNA *in situ *hybridization experiments revealed that 86% (42 of 52) of down-regulated genes in *ewg*^*l1*^mutants, but only 17% (11 of 63) of up-regulated genes in *ewg*^*l1*^mutants, are expressed in the nervous system of late stage embryos (Figure 4c,d; Figures S2 and S3 in Additional data file 1). Interestingly, these RNA *in situ *hybridization experiments further revealed that the vast majority of genes down-regulated in *ewg*^*l1*^mutants are broadly expressed in the entire nervous system, suggesting that they might be direct targets of EWG. From those genes where the RNA expression pattern was analyzed, 30% (22 of 74) of down-regulated genes in *ewg*^*l1*^mutants and 7% (5 of 68) of up-regulated genes in *ewg*^*l1*^mutants did not yield a signal in RNA *in situ *experiments, although they were detected on microarrays at comparable levels. These transcripts might reside, therefore, in tightly packed mRNP particles and not be accessible to *in situ *hybridization after fixation, which has been shown, for example, for Fragile X Mental Retardation Protein (FMRP) mRNA in dendrites [29].

### Analysis of synaptic growth in mutants of genes differentially regulated in *ewg*^*l1*^mutants

Recent efforts in *Drosophila *genome projects have increased the number of mutants to cover 50-60% of all protein coding genes [30-34]. This allows for a functional genomics approach to identify a representative fraction of genes differentially regulated in *ewg*^*l1*^mutants and involved in synaptic growth regulation, and to elucidate general principles that operate in this biological process. We obtained viable mutants for 42% (40 of 95) of genes down-regulated in *ewg*^*l1*^mutants and for 37% (23 of 62) of genes up-regulated in *ewg*^*l1*^mutants (Table S2 in Additional data file 1). Focusing on synaptic growth regulation, we restricted our analysis to genes not involved in basic cellular metabolism unless their expression was enriched in the nervous system. Most of the mutants were *P *element or *piggyBAC *insertions in promotor regions and represent hypomorphic alleles indicated by 38% (21 from 57) showing adult phenotypes (pupal lethality, morphological aberrations, sterility or flightlessness; Table S2 in Additional data file 1) as transheterozygotes over a deficiency or a second allele. A minor portion of mutants was embryonic or early larval lethal (10%, 7 of 70) and could not be analyzed for synaptic growth defects. Viable mutants tend to accumulate genetic modifiers [35]. Therefore, we normalized the genetic background by analyzing transheterozygotes for a chromosomal deficiency, or in some cases transheterozygotes for a second allele. Since the most pronounced effect in *ewg*^*l1*^mutants was at muscle 13, we quantified type 1b boutons at muscle 13.

**Figure 5:**
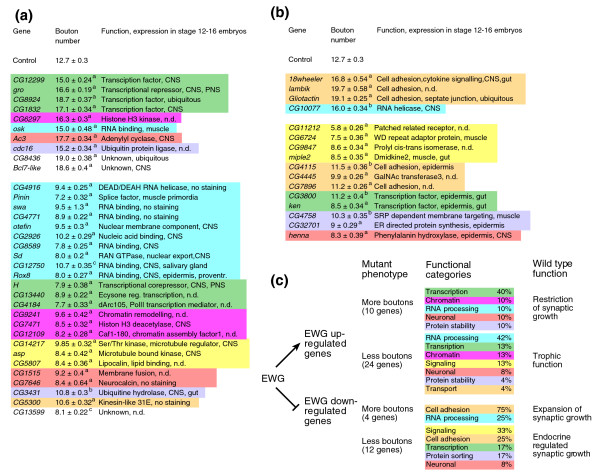
Analysis of synaptic growth in mutants of genes differentially regulated in *ewg*^*l1*^mutants. **(a,b) **Quantification of synaptic growth in mutants of genes differentially regulated in *ewg*^*l1*^mutants. Type 1b boutons at muscle 13 were quantified and are shown as means with standard error (n = 18-22) in transheterozygotes for either a chromosomal deficiency or a second allele from mutants of genes down-regulated (a) or up-regulated (b) in *ewg*^*l1*^mutants that were significantly different (^*a*^*p *≤ 0.0001, ^*b*^*p *≤ 0.001, ^*c*^*p *≤ 0.05) compared to controls (*y w *and *y w *transheterozygous for the corresponding chromosomal deficiency) except for genes involved in basal metabolism. Detailed genotypes with corresponding bouton numbers are listed in Table S2 in Additional data file 1. Genes are clustered according to their function with the color codes used in Figure 4. Note that genes down-regulated in *ewg*^*l1*^mutants are highly enriched for expression in the nervous system (90%) while only a minor portion of genes up-regulated in *ewg*^*l1*^mutants is expressed in the nervous system (36%). **(c) **Summary of mutants in genes differentially regulated in *ewg*^*l1*^mutants with synaptic growth defects and model of EWG regulation of these genes and their roles in synaptic growth regulation. CNS, central nervous system; PNS, peripheral nervous system.

Analysis of synaptic growth in mutants of genes down- and up-regulated in *ewg*^*l1*^mutants revealed that 85% (34 of 40) and 70% (16 of 23), respectively, were associated with statistically significant differences in synaptic growth compared to controls (Figure 5, *y w *line and *y w *transheterozygous for appropriate deficiencies, *p* ≤ 0.0001 for the vast majority; for details, see Table S2 in Additional data file 1). Ninety percent (18 of 20) of the genes down-regulated in *ewg*^*l1*^mutants are also expressed in the nervous system (Figure 5a,c). Clustering these genes into functional classes revealed a strong enrichment for genes involved in the regulation of gene expression (65%, 22 of 34; Figure 5a,c). In contrast, genes up-regulated in *ewg*^*l1*^mutants with synaptic growth defects are mostly not expressed in the nervous system (64%, 7 of 11; Figure 5b,c) and are split into many different functional classes (Figure 5b,c).

For both down- and up-regulated genes in *ewg*^*l1*^mutants with synaptic growth defects, phenotypes could be split into groups with either more or less boutons (Figure 5). Among those genes down-regulated in *ewg*^*l1*^mutants with growth defects in mutants, 29% (10 of 34) had the same overgrowth phenotype as *ewg*^*l1*^mutants (Figure 5a). These results show that EWG-regulated gene expression combines both stimulatory and restrictive functions in synaptic growth regulation, and that restrictive functions dominate, indicated by the overgrowth phenotype of *ewg*^*l1*^mutants.

Based on synaptic growth phenotypes associated with mutants of genes differentially regulated in *ewg*^*l1*^mutants, the following model assigns more distinct roles to these genes in the context of EWG regulation (Figure 5c). Genes down-regulated in *ewg*^*l1*^mutants with an increased number of boutons exert restriction on synaptic growth in the presence of EWG, while those genes down-regulated in *ewg*^*l1*^mutants with a reduced number of boutons in mutants might provide a trophic supply for synaptic growth. For genes up-regulated in *ewg*^*l1*^mutants, overgrowth associated with mutations in these genes is indicative of a role in expansion of synaptic growth as they are repressed in the presence of EWG. This class includes primarily cell adhesion molecules for which a restrictive role has been demonstrated when adhesion has been increased (for example, [36]). Most genes up-regulated in *ewg*^*l1*^mutants with fewer boutons are not expressed in the nervous system. These genes might, therefore, be regulated endocrinologically, indicated by the role of *ewg *in regulating metabolic aspects.

### Erect wing co-regulated genes are functionally related

Pioneering work in yeast has shown that expression of functionally related genes is co-regulated (reviewed in [37]). We therefore used genetic interaction experiments to test for functional connections among genes that are differentially expressed in *ewg *mutants and that are also involved in synaptic growth regulation. Since loss of *ewg *results in more boutons and synaptic overgrowth can be further induced (for example, by overexpression of the *fos *and *jun *heterodimer AP-1, see below), we analyzed double mutants in a representative number of those genes down-regulated in *ewg*^*l1*^mutants with more boutons. For all different combinations tested, none of the double mutant animals had more boutons than the single mutants, indicating that these genes do not act in parallel to regulate synaptic growth as independent effects would be additive (Table S1 in Additional data file 1). Some combinations, however, resulted in significant reduction of bouton numbers (for example,*Ac3*; *Bcl7-like*, *Ac3*; *CG1943*, and *Bcl7-like*; *CG12299*; Table S1 in Additional data file 1), suggesting that a combined loss of function in these genes might affect trophic supply to synaptic growth.

**Figure 6:**
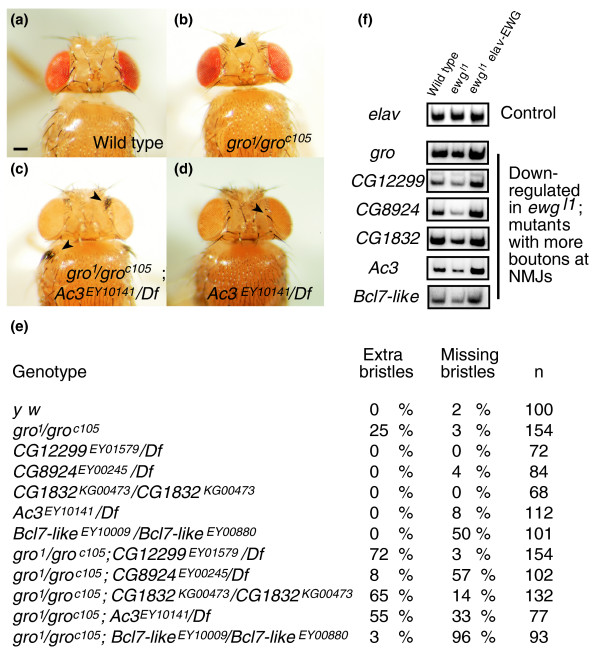
Validation of functional relationships of *ewg *co-regulated genes by genetic interactions. **(a-d) **Top view of head and thorax of wild type, *gro *and *Ac3 *mutants, and *gro Ac3 *double mutants. Note the strong overproliferation of frontal bristle on the head and humeral bristles on the thorax of *gro Ac3 *double mutants compared to *gro *mutants (arrowheads). Some *Ac3 *mutants, as shown in (d), have a reduced number of frontal bristles (arrowhead). Deficiencies used are listed in Table S2 in Additional data file 1. The scale bar in (a) represents 100 μm. **(e) **Analysis of frontal bristle numbers in single and double mutants of genes down-regulated in *ewg*^*l1*^mutants with a synaptic overgrowth phenotype. Note that in all double mutants tested the frontal bristle phenotype is either enhanced or suppressed. Deficiencies used are listed in Table S2 in Additional data file 1. **(f) **Quantitative RT-PCR of genes down-regulated in *ewg*^*l1*^mutants with a synaptic overgrowth phenotype. PCR products using ^32^P labeled forward primers from cycle 26 were analyzed on 6% polyacrylamide gels. *elav*: control (cycle 28).

Next, we validated functional connections among genes down-regulated in *ewg*^*l1*^mutants with a synaptic overgrowth phenotype in another assay. One of the genes in this class is *gro*, for which hypomorphic alleles are known that are associated with an overproliferation of frontal bristles on the head (Figure 6b). *gro *has been described as a transcriptional co-repressor that interacts with a subset of negative transcriptional regulators [38]. We therefore tested a representative number of mutants in genes downregulated in *ewg*^*l1*^mutants in combination with *gro *for a change of the *gro *bristle phenotype. All mutants tested genetically interacted with *gro*, resulting in either an enhancement or suppression of the *gro *bristle phenotype (Figure 6e). None of the single mutants had more bristles, but some had less (Figure 6d), suggesting that the *gro *phenotype can be suppressed (for example, *CG8924 *and *Bcl7-like*), indicated by several roles of *gro *in peripheral nervous system specification [39]. With quantitative RT-PCR, we verified differential expression of these genes in *ewg*^*l1*^mutants and rescue to wild-type expression levels by the presence of an *elav-EWG *transgene in *ewg*^*l1*^mutants (Figure 6f). The genes are also expressed predominantly in the ventral nerve cord (Figure S3 in Additional data file 1; Figure 4c) [40]. Taken together, these data strongly suggest that these genes down-regulated in *ewg*^*l1*^mutants are functionally connected and operate in a common pathway.

### *erect wing* genetically interacts with Notch and Wnt/Wingless signaling in synaptic growth regulation

The predominance of transcriptional and post-transcriptional regulators among genes differentially regulated in *ewg*^*l1*^mutants, together with opposite phenotypes associated with mutants of these genes, suggests that overlapping stimulatory and restrictive pathways are integrated by an extensive regulatory gene network. This model for the regulation of synaptic growth implies that signaling pathways converge in the regulation of gene expression and predicts that *ewg *genetically interacts with several signaling pathways involved in regulating synaptic growth.

Groucho, the protein encoded by *gro*, is differentially regulated in *ewg *mutants and acts in both N and Wg signaling pathways [39,41,42]. Since Wg was previously shown to regulate synaptic growth [43], we first determined if N is also involved in regulating synaptic growth and second, if *ewg *genetically interacts with these two pathways. Therefore, we quantified type Ib bouton numbers at muscle 13 of third instar NMJs in the absence or presence of EWG using the *eFeG *transgene (Figure 1). In this genetic condition, half of the mosaic animals will have one copy of the wild-type *ewg *gene in clones while the other half contains the *ewg*^*l1*^null allele and, therefore, has no EWG protein in clones. Changes in N signaling were achieved through the recombined *eFeG *transgene that expresses GAL4 in the clone and drives either *UAS-N *for N overexpression or *UAS-N-RNAi *for N down-regulation. Expression of *UAS-N-RNAi *in post-mitotic neurons has previously been shown to reduce N levels and to result in long-term memory deficits [11-13]. Changes in *wg *signaling in clones was achieved through overexpression from *UAS *transgenes of *wg *or *pangolin *(*pan*), the transcription factor intracellularly mediating the canonical wingless signal in embryos and wing discs [44-46].

**Figure 7:**
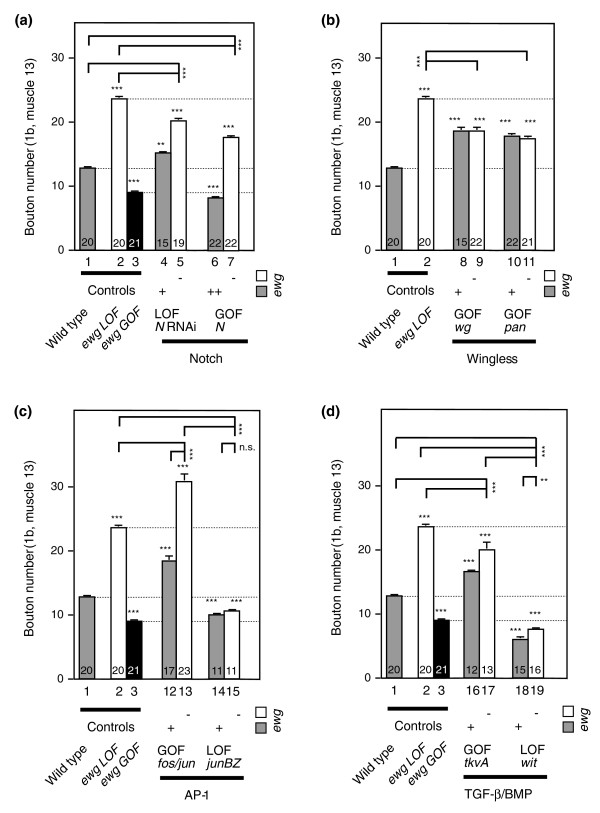
*erect wing *integrates cellular signaling to regulate synaptic growth. **(a-d) **Genetic interaction of *ewg *with known signaling pathways in synaptic growth regulation. Synaptic growth was analyzed at third instar NMJs by quantifying type 1b boutons at muscle 13 of *ewg*^*l1*^*eFeG *clones (white bars and '-' at the bottom of the column) or of *ewg*^*l1*^*eFeG *clones in the presence of an *ewg *wild-type copy (grey bars and '+' at the bottom of the column) in combination with either transgenes for *UAS *constructs or transheterozygous combinations of mutant alleles (*wit*^*A*12^/*wit*^*B*11^) as described for Figure 1. Wild type (bar 1), *ewg *loss of function (LOF, bar 2) and *ewg *gain of function (GOF, black bars, bar 3) were compared to LOF and GOF mutants in Notch (a), Wingless (b), AP-1 (c) and TGF-β/BMP (d) pathways. Overexpression of N is shown in the presence of both *ewg *copies ('++' at the bottom of the column), as one *ewg *copy in the presence of excess N does not result in a significant increase of bouton numbers compared to wild type. For both *N *and *tkvA*, two copies of *UAS *transgenes were used; a single copy did not significantly alter bouton numbers. Shown are means of bouton numbers with standard errors (n = 11-23, numbers at the bottom of bars). Bars are numbered below the x-axis. Statistical significance of differences from comparisons with wild type is shown on top of bars (***p < 0.0001, ** *p *< 0.005, n.s. for non significant). Other relevant comparisons are marked by horizontal bars with the statistical significance indicated on the side.

Similar to *ewg*, N restricts synaptic growth, resulting in increased bouton numbers indicated by down-regulation of *N *with RNA interference during larval life (from two independent *UAS *inserts, *p* = 0.005 for bar 1 compared with bar 4, Figure 7a) or in reduced bouton numbers when N was overexpressed (*p *< 0.0001 for bar 1 compared with bar 6, Figure 7a) compared to wild-type control.

In the absence of EWG, removing N (*N *LOF) resulted in intermediate numbers of boutons compared to wild type and *ewg *LOF (*p *< 0.0001 for bar 5 compared with bar 1 and 2, Figure 7a). The increase in bouton numbers in *N *LOF (bar 5 compared with bar 2) is not additive to the numbers observed in the absence of EWG (bar 2 compared to bar 1, as seen for AP-1 overexpression, see below), suggesting that regulation of synaptic growth by N is not independent of *ewg *and indicating that part of the N signal is required for the full effect seen in *ewg *LOF. Overexpression of N (*N *GOF) in the absence of EWG resulted in intermediate numbers of boutons compared to wild type and *ewg *LOF (*p *< 0.0001 for bar 7 compared with bars 1 and 2, Figure 7a).

Overexpression of *wg *increased bouton numbers (*p *< 0.0001 for bar 1 compared with bar 8, Figure 7b), as previously observed [43]. Overexpression of *pan *also resulted in increased bouton numbers (*p *< 0.0001 for bar 1 compared with bar 10, Figure 7b), demonstrating that the canonical *wg *pathway operates presynaptically to stimulate synaptic growth.

In the absence of EWG, overexpression of both *wg *and *pan *were epistatic to *ewg *LOF and bouton numbers were significantly reduced compared to *ewg *LOF (*p *≤ 0.0001 for bar 2 compared with bars 9 and 11, Figure 7b).

### AP-1 and TGF-β act together with *erect wing *in synaptic growth regulation

The *fos *and *jun *heterodimer AP-1, and TGF-β signaling comprise two other known pathways involved in regulating synaptic growth [4,5,7,8]. Both AP-1 and TGF-β stimulate synaptic growth compared to wild type as evident either by overexpression of *fos *and *jun *together (*p *< 0.0001 for bar 1 compared with bar 12, Figure 7c) and an activated BMP type I receptor (*tkvA*, from two copies of *UAS-tkvA*, *p *< 0.0001 for bar 1 compared with bar 16, Figure 7d), or by removal of AP-1 activity through overexpression of a dominant negative form of *jun *(*junBZ*, *p *< 0.0001 for bar 1 compared with bar 14, Figure 7c), or in the BMP type II receptor mutant *wishful thinking *(*wit*, *p *< 0.0001 for bar 1 compared with bar 18, Figure 7d), which is largely in agreement with previous observations [4,5,7,8]. Overexpression of either *fos *or *jun *alone has no effect on synaptic growth [4] (and data not shown).

Next, we wanted to define how these two pathways relate to *ewg *mediated regulation of synaptic growth. In the absence of EWG, overexpression of AP-1 is additive and further increases bouton numbers significantly (*p *< 0.0001 for bar 13 compared with bars 2 and 12, Figure 7c). Inhibiting AP-1 function by expressing the dominant negative *junBZ *completely removes the stimulatory effect of *ewg *LOF (*p *< 0.0001 for bar 15 compared with bars 2 and 13, and non-significant for bar 15 compared to bar 14, Figure 7c), suggesting that AP-1 functions downstream of *ewg*.

Overexpression of *tkvA *(from two copies of *UAS-tkvA*) in *ewg *LOF resulted in intermediate numbers of boutons compared to wild type and *ewg *LOF (*p *< 0.0001 for bar 17 compared with bars 1 and 2, Figure 7d), indicating that the TGF-β signaling pathway does not act independent of *ewg *in synaptic growth regulation. Removal of the BMP type II receptor *wit *significantly reduced bouton numbers in *ewg *LOF (*p *< 0.0001 for bar 19 compared with bars 2 and 17, Figure 7d). The strong reduction of bouton numbers in *wit *mutants in *ewg *LOF compared to wild type (*p *< 0.0001 for bar 19 compared with bar 1, Figure 7d) further suggests that TGF-β signaling also includes a component that acts downstream of *ewg*.

## Discussion

Several pathways have been identified that stimulate synaptic growth at NMJs of *Drosophila *larvae (Wnt/Wingless, TGF-β/BMP and *jun *kinase) [2-4]. Overexpression of AP-1 [4] and mutants in regulatory genes involved in Wnt/Wingless and TGF-β/BMP pathways (*spinster*, *highwire*, *shaggy *and the proteasome) [9,47-49] can increase bouton numbers, suggesting that synaptic growth is regulated through the balance of stimulatory and restrictive signals. Here, we have identified such a restrictive role for the transcription factor EWG and, through the analysis of EWG-regulated genes, for the N pathway in the regulation of synaptic growth. Using genetic mosaics, we further demonstrate that EWG's role in synaptic growth regulation is cell-autonomous, suggesting that the transcriptional regulator EWG mediates this restrictive effect through the alteration of transcription pre-synaptically.

Analysis of genes differentially expressed in *ewg*^*l1*^mutants revealed a rather unexpected set of genes involved in synaptic growth regulation, besides an expected number of metabolic genes due to homology of EWG to human NRF-1 [50]. Most genes that could account for the phenotype of *ewg*^*l1*^mutants, and that are thus expressed in the nervous system, are involved in transcriptional and post-transcriptional regulation of gene expression. Although changes of transcript levels in *ewg*^*l1*^mutants were mostly moderate, their significance was validated through mRNA profiling with rescued *ewg*^*l1*^mutants under the same conditions of RNA preparation and microarray hybridization. In addition, differences in gene expression in *ewg*^*l1*^mutants were validated using quantitative RT-PCR and biochemical assays with regard to predicted changes in glycogen levels based on differential regulation of genes involved in gluconeogenesis (for example, Pepck; Additional data file 1). Furthermore, genetic interaction experiments in double mutants with increased bouton numbers support that these co-regulated genes are functionally connected in regulating synaptic growth.

The group of neuronal genes among those differentially regulated in *ewg*^*l1*^mutants that have been demonstrated to have roles in synaptic growth or could account for it, is remarkably small. In particular, from the large number of cell adhesion molecules and cytoskeletal proteins present in the *Drosophila *genome only a handful is differentially regulated. Similar results have also been obtained in response to JNK and AP-1 signaling [15]. These results are in contrast to changes in gene expression induced by acute or chronically enhanced neuronal activity in *Drosophila *seizure mutants, which also result in synaptic overgrowth [51]. Here, the vast majority of differentially regulated genes are for cell adhesion molecules and cytoskeletal proteins or their regulators, and genes involved in synaptic transmission and neuronal excitability; transcriptional or post-transcriptional regulators comprise only a minor portion. These differences could be explained by separate pathways regulating growth independent of neuronal activity [36,52] (and this study).

Particularly striking is the large number of genes involved in RNA processing among genes differentially expressed in *ewg*^*l1*^mutants. Although local regulation of gene expression is required in growth cones of navigating axons, a prominent role for pre-synaptic regulation of gene expression at the RNA level is only just emerging [53], but is a hallmark of post-synaptic plasticity [29,54]. Several RNA binding proteins have been implicated in memory storage [23,55,56]. *osk *and *CPSF *(cleavage and polyadenylation specificity factor) are among the genes differentially regulated in *ewg*^*l1*^mutants. Other genes involved in RNA processing differentially regulated in *ewg*^*l1*^mutants comprise the whole spectrum of regulation at the post-transcriptional level, from nuclear organization (*otefin*), alternative pre-mRNA processing (*Pinin*, *CPSF*, *Rox8*) and export/import *(Segregetion distorter*, *Nxf2*, *CG11092*, *Karyopherin*, *Transportin*) to transport, localization and translation (*oskar*, *swallow*, *ribosomal protein genes S5 *and *Rpl24*), and likely also include the regulation of mRNA stability (*Rox8*).

An intriguing connection between *ewg *and signaling pathways involved in regulating synaptic growth is indicated by differentially regulated components of the Wg and N pathways (*gro *and *Hairless*) [39,41,42,57] in *ewg*^*l1*^mutants. Consistent with a role of the co-repressor *gro *in Wg and N mediated transcriptional regulation of synaptic growth, Wg and N signaling pathways do not operate independently of *ewg *in genetic interaction experiments. The transcriptional regulatory networks of EWG, Wg and N seem to be highly interwoven. Overexpression of *pan*, the transcriptional mediator of canonical Wg signaling, which is repressed by *gro*, does not lead to a further expansion of synaptic growth in *ewg *mutants, suggesting a requirement for *ewg*-regulated genes. This effect could be mediated by deregulated N signaling, which is also repressed by *gro*, but antagonistic to Wg in synaptic growth. Thus, removal of *gro*, as in *ewg*, will relieve the repressive effect of N and antagonize the stimulatory effect of *pan*. In the complementary situation, removal of N increased bouton numbers further in the absence of EWG, which is consistent with an increase in Wg signaling as a result of down-regulated *gro *in *ewg *mutants. Antagonism between N and Wg pathways has also been found in wing discs, where N inhibits *armadillo *(*arm*), the transcriptional co-activator of canonical Wg signaling [58]. Intriguingly, *gro *has also been found to be a target of receptor tyrosine kinase signaling and, thus, can combine additional pathways with N and Wg signaling [59,60]. In addition to transcriptional hierarchies, chromatin remodeling has also been implicated in synaptic plasticity [61]. Strikingly, *CG6297*, a *Drosophila *homologue of the histone deacetylase RPD3, is differentially expressed in *ewg*^*l1*^mutants and physically interacts with *gro *[62].

How *ewg *exerts its effect on TGF-β signaling is less clear. A prominent regulatory step in this pathway is the regulated degradation of the SMAD co-factor Medea by Highwire [5]. Several genes involved in regulating protein stability are differentially down-regulated in *ewg *mutants (*CG6759*, *CG3431*, *CG4973*, *CG7288*, *CG3455*, *CG9327 *and *CG9556*). Lower expression levels of these genes might interfere with stabilization of Medea and explain why the effect of activated TGF-β signaling is not additive in the absence of EWG (*tkvA *GOF *ewg *LOF). Bouton numbers in *wit *null mutants are marginally increased in the absence of EWG, suggesting further that genes regulated by SMADs are involved in mediating synaptic overgrowth in *ewg*^*l1*^mutants. Potentially, *ewg *could also regulate TGF-β signaling through the endosomal pathway involving spinster and/or spichthyin [9,63].

Functionally related genes have been shown to be co-regulated [37], suggesting additional ELAV targets in EWG-regulated gene networks. Indeed, ELAV negatively regulates alternative splicing of the penultimate exon in *armadillo *(*arm*) [19]. Exclusion of this exon, which truncates the carboxyl terminus of *arm*, reduces Wg signal transduction, which is in agreement with *ewg*'s antagonistic role relative to Wg signaling. Another known ELAV target gene is *neuroglian *(*nrg*), where a role in synapse formation has recently been demonstrated in the giant fiber system [64]. Taken together, the establishment of a gene network regulated by EWG will now serve as valuable tool to identify further ELAV regulated modules that shape the synapse.

## Conclusion

The transcription factor EWG is a major target of the RNA binding protein ELAV, which regulates EWG protein expression via a splicing mechanism. EWG is required pre-synaptically and cell-autonomously at third instar neuromuscular junctions to restrict synaptic growth, demonstrating that restrictive activities at gene expression levels are also required for synaptic growth regulation. EWG mediates regulation of synaptic growth primarily by increasing transcript levels of genes involved in transcriptional and post-transcriptional regulation of gene expression. Genes at the end of the gene expression hierarchy (effector genes) represent only a minor portion of EWG-regulated genes. Since analysis of mutants in genes differentially regulated in *ewg*^*l1*^mutants revealed that these genes are involved in both stimulatory and restrictive pathways of synaptic growth, and since *ewg *genetically interacts with a number of signaling pathways (Wingless, Notch, TGF-β and AP-1), our results suggest that synaptic growth in *Drosophila *is regulated by the interplay of multiple signaling pathways rather than through independent pathways.

## Materials and methods

### Fly genetics, recombinant DNA technology and microarrays

Fly breeding, genetics and P-element mediated transformation of *Drosophila*, and recombinant DNA technology were done according to standard procedures as detailed in Soller and White [21] and Soller *et al*. [65]. In all experiments the *ewg *NS isoform was used [24]. The *eFeG *construct *P{w+ = elav-FRT-EWG-FRT-GAL4*} is basically the same as the *EWG*^*NS *^construct [25] except that the *ewg *cDNA is flanked by FRT sites followed by the coding sequence of the yeast GAL4 transcription factor.

For cloning of the *eFeG *construct, the 3.5 kb *elav *promoter [25] was cloned into a linker modified *CaSpeR 4 *(*Drosophila *Genomics Resource Center, Bloomington, IN, USA) with a blunt *Eco*RI site and *Not*I site to generate the construct *C4MMelav2*. The 5' FRT site was added to the *ewg *gene by nested PCR with primers FRTewgF1 (AGAAAGTATAGGAACTTCAGAGCGCTTTTGAAGCTAgcccaccgccaaactggccaccacaagc; the *ewg *sequence is indicated in small letters and restriction sites are underlined) and FRTewgF2 (CGGGGATCTTGAAGTTCCTATTCCGAAGTTCCTATTCTCTAGAAAGTATAGGAACTTCAGAGCGC) and ewg3cR1Spe (GGAACTAGTCAACACCTTGAACCTGGGCAGTTGTACCATCC) and cloned into *C4MMelav2 *with a blunt *Not*I site and *Spe*I site to generate *C4MMelavFRTewg*. For cloning of the 3' part of *eFeG*, a *Hpa*I-*Eco*RI fragment encoding the 3' part of the *ewg *cDNA was cloned into *pBluescript *(Stratagene, Cedar Creek, TX, USA) cut with *Eco*RV and *Eco*RI to generate *SC3HE*. Subsequently, the 3' FRT site was cloned into *SC3HE *cut with *Eco*RI and *Sac*II with two oligonucleotides (FRT-A *Eco*RI x *Not*I, *Spe*I, *Sac*II: AATTCCGGGGATCTTGAAGTTCCTATTCCGAAGTTCCTATTCTCTAGAAAGTATAGGAACTTCAGAGCGCTTTTGAAGCTGCGGCCGCACTAGTGC and FRT-B:ACTAGTGCGGCCGCAGCTTCAAAAGCGCTCTGAAGTTCCTATACTTTCTAGAGAATAGGAACTTCGGAATAGGAACTTCAAGATCCCCGG) after the *ewg *3' untranslated region (UTR) to generate *SC3HE FRT*. GAL4 with the *hsp70 *3' UTR from *pGaTN *(*Drosophila *Genomics Resource Center) was cloned with *Not*I and *Spe*I into *SC3HE FRT *to generate *eFG*. From *eFG*, the 3' part of *ewg*, the FRT site and the GAL were cloned with *Acc*65I and *Spe*I into *C4MMelavFRTewg *to generate the *eFeG *construct.

For the analysis of synaptic growth in EWG deficient neurons, two independent inserts of the *eFeG *construct on the X and third chromosomes that fully rescue *ewg*^*l1*^mutants were used. To induce clones with *eFeG *transgenes, 14-18 h old embryos from *ewg*^*l1*^*eFeG *females crossed with males carrying *hs-flp UAS-CD8::GFP (P{hsFLP}38*, *P{UAS-mCD8::GFP.L}LL5 *[66,67]) on the second chromosome were heat shocked for 45 minutes at 35°C, rested for 45 minutes at room temperature and incubated for another 45 minutes at 37°C. For genetic interaction experiments, the *ewg*^*l1*^*eFeG *and *hs-flp UAS-CD8::GFP *chromosomes were combined with the following *UAS *transgenes or mutants (from Bloomington stock center, Bloomington, IN, USA) or as noted: *UAS-wg *(second or third, *P{w [+mC] = UAS-wg.H.T:HA1}6C *and *P{w [+mC] = UAS-wg.H.T:HA1}3C *[45]), *UAS-pan *(second or third, *P{w [+mC] = UAS-pan.dTCF}24 *and *P{w [+mC] = UAS-pan.dTCF}4 *[45]), *UAS-N-RNAi*_*i *_(second and/or third, *P{w [+mC] = UAS-N.dsRNA.P}14A *and *P{w [+mC] = UAS-N.dsRNA.P}9G *[13]), *UAS-N *(second and/or third, gift form S Artavanis-Tsakonas [68]), *UAS-tkvA *(second and/or third, gift from M O'Connor [7]), *UAS-junBZ *(second or third, *P{Jbz}1 *and *P{Jbz}10 *[69]), *UAS-fos *(*P{UAS-Fra}*, second [69]), *UAS-jun *(*P{UAS-Jra}2*, third [69]) and *wit*^*A*12 ^with *wit*^*B*11 ^[8], UAS-Tetanustoxin (gift from S Sweeney, *P{UAS-TeTxLC.tnt}*, CNT-E, second [70]). Overexpression of *UAS-wg*, *UAS-pan*, *UAS-N-RNAi *and *UAS-N *with *elav-GS-GAL4 *(third, *P{elav-Switch.O} *[27]) resulted in significant changes in bouton numbers comparable with overexpression with recombined *eFeG *compared to wild type.

The *elav-NRF-1 *construct was made by exchanging the *ewg *open reading frame (ORF) in *elav-EWG *with the ORF of NRF-1 as follows. The NRF-1 ORF was amplified by PCR with primers NRF-F1 (TAGAGCGGCCGCTCGAGAATTCtttacgtggtcctttatttg) and NRF-R1 (CATGCCTTCTATGGGCTCCAgTCACTGTTCCAATGTCACCACCTC) from a cDNA clone (gift from R Scarpulla [18]), cut with *Not*I and combined with the 3' UTR of *ewg *amplified by PCR with primers ewgUTR-F1 (ctggagcccatagaaggcatg) and M13rev (GGAAACAGCTATGACCATG) from the *ewg *cDNA clone in pBluscript SK^+ ^cut with *Spe*I and with the *C4MMelav2 *cut with *Not*I and *Spe*I in a three way ligation to generate *C4MMelav2NRF-1*. NRF-1 expression was verified in transgenes by western blots. Inserts of *elav-NRF-1 *on the second and third chromosomes were used. *elav-ELAV *is the *elav *ORF under the control of the endogenous promoter followed by an α-tubulin 3' UTR, and the *elav-NRF-1 *insert on the second chromosome was used [25]. For the *UAS-ewg *construct, the NS isoform (*EWG*^*NS*^) including the *ewg *3' UTR [25] was cloned with *Bam*HI and *Spe*I into a modified *pUAST *[71] cut with *Bgl*II and *Spe*I where the SV40 3' UTR had been removed. Conditional overexpression of EWG from *UAS-ewg *(third) with *elav-GS-GAL4 *(*elav-GeneSwitch-GAL4 *[27]) (third) was induced by adding RU486 (Mifepristone, Sigma, St Louis, MI, USA) in 100 μl of 50% ethanol to larvae two days before dissection [27]. The *elav *alleles used were *elav*^*ts1*^, a temperature sensitive allele, and *elav*^*e5*^, a null allele [71]. Mutants used in genes differentially regulated in *ewg*^*l1*^mutants are listed in Table S2 in Additional data file 1 and were obtained from Bloomington, Harvard and Tübingen stock centers as indicated in Flybase [72].

Embryos for microarray analysis were produced by crossing *ewg*^*l1*^/*C155-GAL4*; *UAS-GFP*/+ females to *C155-GAL4; UAS-GFP *males (*P{GawB}elav*^*C*155^; *P{UAS-GFP.S65T}T2 *[65,73]). The X-chromosomal *C155-GAL4 *enhancer trap was inserted in the neighboring *elav *gene and drives GFP expression in neurons and neuroblast [65]. From the progeny of this cross, non-GFP expressing embryos, which are male, were then hand-picked 16-18 h after egg laying and RNA was extracted from pools of 50 embryos with Trizol (Invitrogen, Carlsbad, CA, USA). Pooled RNA from a total of 300 embryos was then reverse-transcribed with an oligo-dT primer containing a T7 promoter and, after the synthesis of the second strand, linearly amplified by *in vitro *transcription with T7 RNA polymerase and labeled with biotinylated NTPs according to the manufacturers instructions (Genechip Protocol for Eukaryotic Target preparation, Affymetrix, Santa Clara, CA, USA) yielding between 20 and 50 μg of RNA. Amplified RNA (15 μg) including spike controls was hybridized to Affymetrix *Drosophila *genome arrays Version X using the Affymetrix Gene Chip Instrument System overnight at 42°C. Arrays were washed and stained with streptavidin-phycoerythrin before scanning on an Affymetrix Gene Chip scanner.

Quantitative RT-PCR was done with 5' ^32^P labeled forward primers and PCR products from unsaturated cycles (24, 26, 28 or 30 depending on expression levels of a particular gene) were analyzed on 6% polyacrylamide gels essentially as previously described [19]. Primer sequences are available upon request.

### Microarray data analysis

Gene chip data were analyzed using the software packages MAS version 5 (Affymetrix) and dChip. Scanned data were first processed with MAS to convert raw image files (.DAT) to probe signal values files (.Cel). Probe signal files were normalized across samples using dChip invariant set method. Summary values for each probe set were calculated with PM-only model in dChip. Microarray data have been deposited in MIAMExpress [74] under the accession number E-MEXP-1312.

### Western blotting, RNA *in situ *hybridization, immunostaining and statistics

Western blotting was done as previously described [24]. Quantification of western blots was done with Quantity ONE 4.2.3 (Bio-Rad, Hercules, CA, USA). RNA *in situ *hybridizations were done according to the BDGP protocol, or obtained from the BDGP web page [75]. For the analysis of NMJs, wandering third instar larvae were dissected in phosphate-buffered saline and fixed with 4% formaldehyde for 30 minutes. The following antibodies were used: anti-DSYT (1:200; gift from T Littelton [26]), anti-DLG (1:100; gift from V Budnik [76]), anti-CSP (1:100; gift from K Zinsmaier [77]), anti-Nc82 (1:6; Developmental Studies Hybridoma Bank), anti-Highwire (1:6; Developmental Studies Hybridoma Bank), Mab22C10 (1:200; Developmental Studies Hybridoma Bank), FITC conjugated anti-HRP (1:200; Cappel, Cochranville, PA, USA), FITC conjugated anti-CD8 (1:200; Cappel). FITC, TRITC (Jackson Labs, Bar Harbor, MA, USA) and Cascade Blue (Molecular Probes, Eugene, OR, USA) conjugated secondary antibodies were used 1:200. Confocal images were acquired at 18°C using a Leica TCS SP2 confocal scanning microscope with a Plan APO HC 10 × 0.4 numerical aperture objective for eye discs, or under oil with a Plan APO HCX 63 × 1.4 numerical aperture (NMJs) or a Plan APO HCX 100 × 1.4 numerical aperture (single boutons) objective and Leica software. Image files were converted to TIFF and merged using Photoshop CS2 (Adobe). Levels of individual channels were adjusted where applicable to maximize pixel range. Flies were photographed with a digital camera (Nikon) on a Leica Stereoscope at 3× magnification. Statistical analysis of bouton numbers was done with ANOVA followed by *post hoc *analysis with Fisher's PLSD (protected least significant difference) for multiple comparisons, or with *t*-test for two samples, using StatView.

## Abbreviations

BDGP, Berkeley *Drosophila *Genome Project; BMP, bone morphogenetic protein; GFP, green fluorescent protein; LOF, loss of function; N, Notch; NMJ, neuromuscular junction; ORF, open reading frame; TGF, transforming growth factor; UTR, untranslated region.

## Authors' contributions

IH and MS conceived the work, and designed and performed the experiments. KW gave advice and helped with the interpretation of results and writing of the paper. MS wrote the paper.

## Additional data files

The following additional data are available with the online version of this paper. Additional data file 1 includes supplemental materials and methods, Figures S1-S3 and Tables S1-S3.

## Supplementary Material

Additional data file 1 Figure S1 shows additional experiments examining *ewg*'s role in the regulation of basic metabolism and mitochondrial proliferation. This also contains supplemental materials and methods. Figure S2 shows representative RNA *in situ *hybridization experiments with genes differentially regulated in *ewg*^*l1*^mutants. Figure S3 shows RNA *in situ *hybridization experiments with genes down-regulated in *ewg*^*l1*^mutants. Table S1 lists bouton numbers in double mutants of genes differentially regulated in *ewg*^*l1*^mutants with a synaptic overgrowth phenotype. Table S2 lists genotypes of mutants of genes differentially regulated in *ewg*^*l1*^mutants and their bouton numbers. Table S3 lists the fold change of genes differentially regulated in *ewg*^*l1*^mutants and rescued by *elav-EWG*. Click here for file
