# Supplementary material for: Erect wing regulates synaptic growth in Drosophila by integration of multiple signaling pathways
Source: Genome Biol. 2008 Apr 17;9(4):R73. doi: 10.1186/gb-2008-9-4-r73 (PMC2643944; doi:10.1186/gb-2008-9-4-r73)
Supplement: Additional data file 1 — Figure S1 shows additional experiments examining ewg's role in the regulation of basic metabolism and mitochondrial proliferation. This also contains supplemental materials and methods. Figure S2 shows representative RNA in situ hybridization experiments with genes differentially regulated in ewgl1mutants. Figure S3 shows RNA in situ hybridization experiments with genes down-regulated in ewgl1mutants. Table S1 lists bouton numbers in double mutants of genes differentially regulated in ewgl1mutants with a synaptic overgrowth phenotype. Table S2 lists genotypes of mutants of genes differentially regulated in ewgl1mutants and their bouton numbers. Table S3 lists the fold change of genes differentially regulated in ewgl1mutants and rescued by elav-EWG. [file gb-2008-9-4-r73-S1.pdf]

Supplementary Material

Erect wing regulated metabolic pathways

The human homologue of EWG, NRF-1, had previously been implicated in mitochondrial biogenesis and various metabolic pathways [78], that could have accounted for the observed synaptic overgrowth phenotype [79, 80]. Genes differentially regulated in ewg^{l1} mutants, however, were not enriched in mitochondrial genes and genes involved in mitochondrial biogenesis. Also, delivery of mitochondria to synapses was not affected (Fig S1). Several genes differentially regulated in ewg^{l1} mutants involved in basic metabolism are assigned roles in energy metabolism through the generation of ATP by inhibiting gluconeogenesis and mobilizing lipid stores (*CG5171*, *CG10611*, Pepck, *CG3752*, *CG7176*, *CG9186*, *CG7279*, *CG33174*). Analysis of ATP levels in late embryos did not reveal a significant difference (13.5±2.2 pMol/embryo in ewg^{l1} vs 14.4±3.1 pMol/embryo in wild type, mean±SE, n=9). In addition, reduced ATP levels and concomitant reduction of neuronal activity do not affect bouton numbers [81]. To biochemically confirm the inhibitory role of EWG in gluconeogenesis indicated from the expression profiling data, we measured glycogen and total lipid levels in ewg^{l1} embryos and compared them with wild type embryos. Glycogen levels were significantly increased in ewg^{l1} embryos (p=0.04, 211±16.7 ng/embryo in ewg^{l1} (n=11) vs 156±17.9 ng/embryo in wild type (n=7), mean±SE, p<0.05), while differences in total lipid levels (728±35 ng/embryo in ewg^{l1} (n=11) vs 745±52 ng/embryo in wild type (n=7), mean±SE) were not significantly different due to the higher amount of lipid present per embryo and the much higher amount of energy stored in lipids. Other co-regulated metabolic pathways were found to be

involved in NADPH regeneration (*CG3523*, *CG31522*, *CG2781*), ethanol metabolism (*CG9390*, *CG3752*, *Adh*), or have been implicated in O_2 sensing (*osiris* gene cluster, [82]).

Supplementary Materials and Methods

ATP levels in pools of 10 embryos were measured by luminescence of luciferase that produces light in proportion to ATP levels according to the manufacturers instructions (Promega). For total lipid and glycogen measurements 10 embryos were fixed in 50 μl ethanol at 90° C for 5 min, homogenized in 100 μl 2% $NaSO_4$. Then 900 μl chloroform/methanol (1:1) was added. After vigorous vortexing, Glycogen was pelleted and 200 μl from the lower phase was taken for total lipid analysis. The glycogen pellet was then dissolved in 30% KOH and heated for 10 min at 95° C to inactivate residual oligosacharides. Cuticular debris was removed by centrifugation and glycogen was pelleted by adding 700μl ethanol and ethanol washed twice. Detection of total lipids and glycogen by vanilin and anthron reactions was as described [83]. Samples with values below 50%, or above 200% of the mean were excluded from the analysis.

Figure S1. Mitochondrial biogenesis and distribution is normal in *ewg^{l1}* mutants.

A-F) Mitochondrial distribution in synapses of *ewg^{l1}* mutants. NMJs were stained with anti-SYT (A and D) or with an antibody against mitochondria localized GFP expressed from *UAS-mitoGFP* by a recombined *eFeG* transgene in wild type or *ewg^{l1}* mosaics.

G-J) Mitochondrial distribution in photoreceptor neurons of *ewg^{l1}* mutants. Mitochondria were stained with an antibody against mitochondrial localized GFP expressed from *UAS-mitoGFP*

by a recombined *eFeG* transgene in wild type or *ewg*^{l1} mosaics. Nuclei in H and J were stained with DAPI.

Figure S2. Expression patterns of genes differentially expressed in *ewg*^{l1} mutants.

A and B) Expression patterns in late stage embryos of genes differentially downregulated (A) or upregulated (B) of in *ewg*^{l1} mutants.

Figure S3. Expression pattern of genes downregulated in *ewg*^{l1} mutants with a synaptic overgrowth phenotype in mutants.

Table S1. Bouton numbers in double mutants with a synaptic overgrowth phenotype of genes differentially expressed in *ewg*^{l1} mutants.

Table S2. Bouton numbers in mutants of genes differentially expressed in *ewg*^{l1} mutants.

Table S3. Fold change of genes differentially regulated in *ewg*^{l1} mutants and rescued by *elav-EWG*.

Figure S1

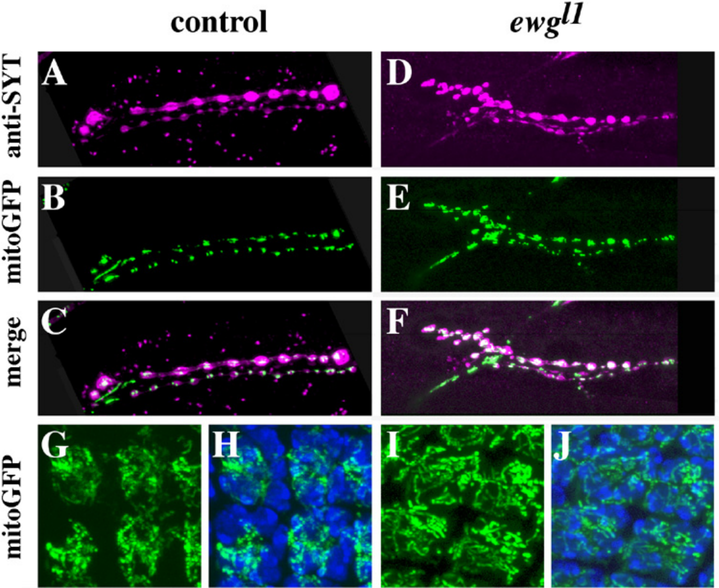

Figure S2

A

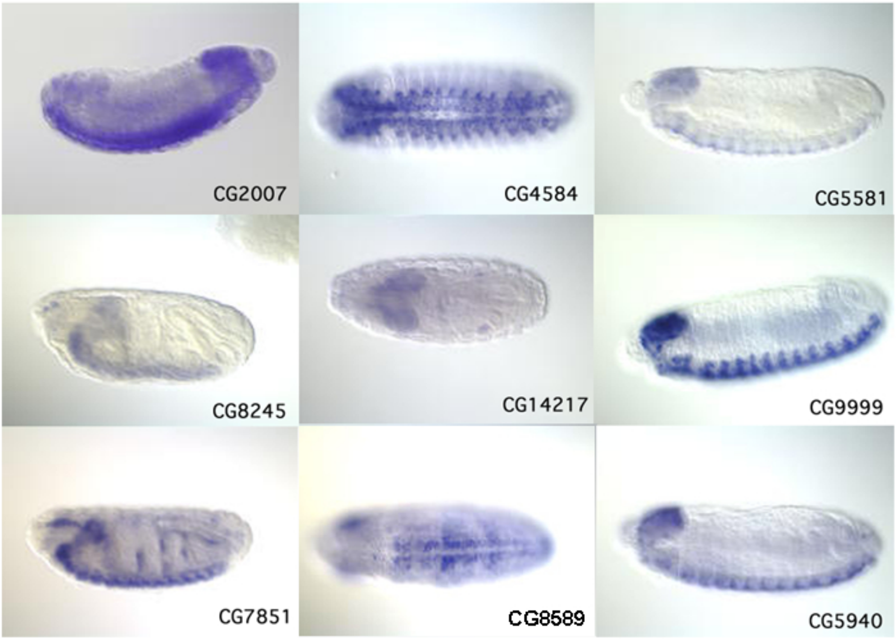
CG2007
CG4584
CG5581
CG8245
CG14217
CG9999
CG7851
CG8589
CG5940

B

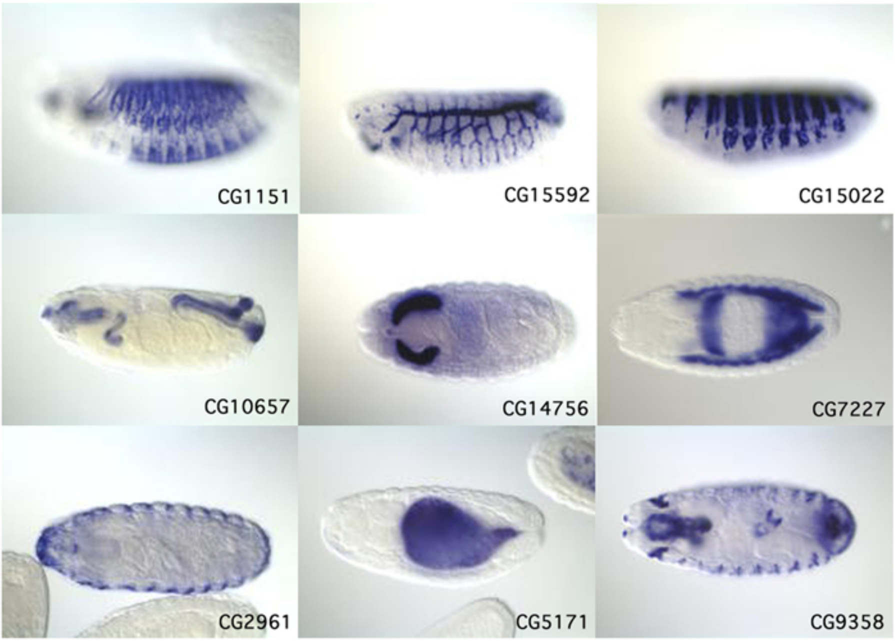
CG1151
CG15592
CG15022
CG10657
CG14756
CG7227
CG2961
CG5171
CG9358

Fig S3

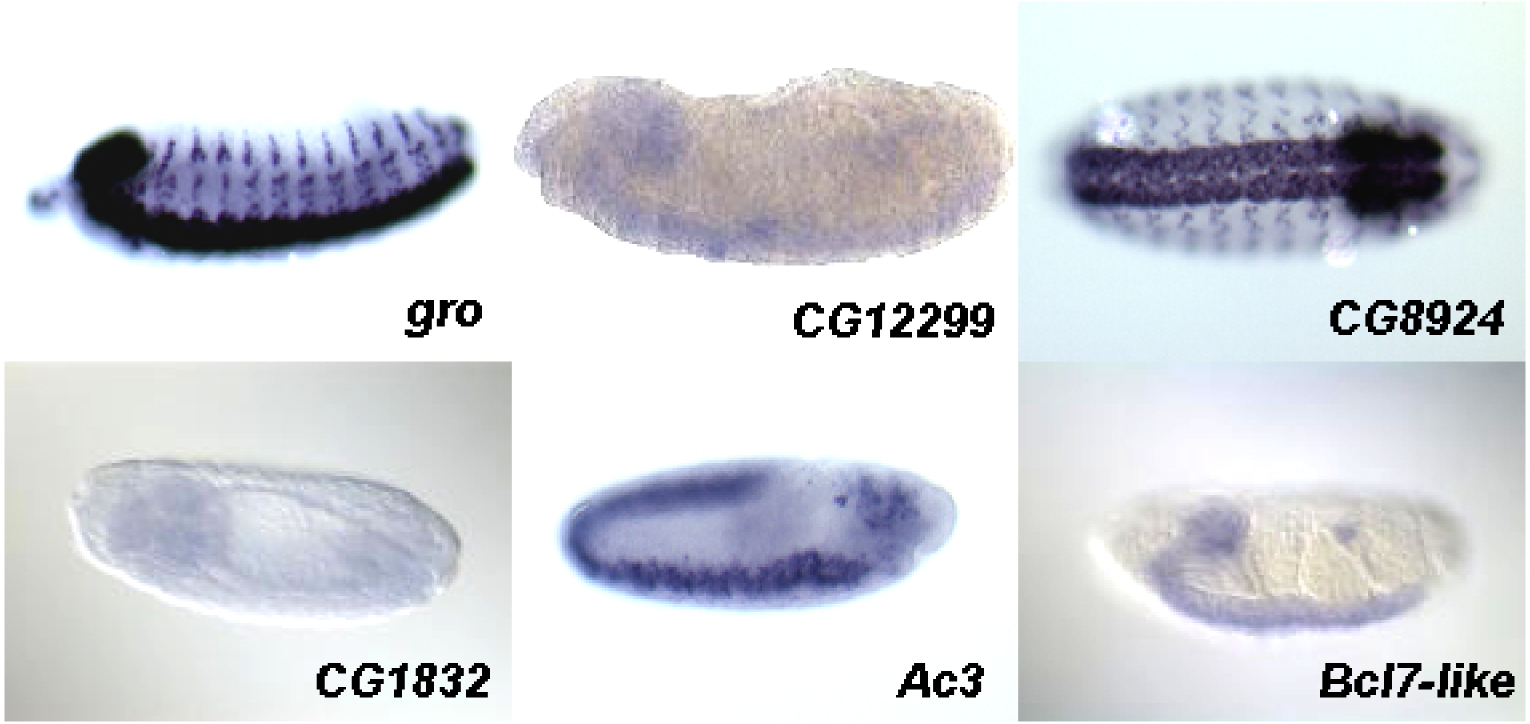

Table1: Synaptic growth in double mutants of *ewg* differentially regulated genes

Genes	genotype of mutants	Bouton number (Type 1b, muscle 13)	
Control		12.7 ± 0.3	(20)
gro	gro^{1}/gro^{C105}	16.6 ± 0.19	(21) [a]
Ac3	P{EPgy2}Ac3^{EY10141}/Df(2L)Exel7081	17.35 ± 0.57	(20) [a]
Bcl-7like	Df(1)9A4-5/P{EPgy2}BCL7-like^{EY10009}	18.6 ± 0.44	(20) [a]
CG12299	P{EPgy2}CG12299^{EY01579}/Df(2L)Exel7820	15.0 ± 0.24	(23) [a]
CG8924	Df(1)sd72b/P{EPgy2}CG8924^{EY00245}	18.7 ± 0.37	(21) [a]
CG1832	P{SUPor-P}CG1832^{KG00473}/P{SUPor-P}CG1832^{KG00473}	17.1 ± 0.34	(22) [a]
CG6297	P{SUPor-P}JIL-1^{KG02848} /P{SUPor-P}JIL-1^{KG08229}	16.3± 0.3	(22)[a]
CG1943	P{EPgy2}CG1943^{EY04914}	18.2 ± 0.26	(24) [a]
CG8436	Df(3R)BSC24/P{EPgy2}EY05475	19 ± 0.38	(21)[a]
gro; CG12299	P{EPgy2}CG12299^{EY01579}/Df(2L)Exel7820; gro^{1}/gro^{C105}	11.9 ± 0.46	(28)[b]
gro; CG8924	Df(1)sd72b/P{EPgy2}CG8924^{EY00245}; gro^{1}/gro^{C105}	18 ± 0.47	(21) [a]
gro; CG1832	P{SUPor-P}CG1832^{KG00473}/P{SUPor-P}CG1832^{KG00473}; gro^{1}/gro^{C105}	19.1 ± 0.5	(21) [a]
gro; Ac3	P{EPgy2}Ac3^{EY10141}/Df(2L)Exel7081; gro^{1}/gro^{C105}	15.0 ± 0.39	(20) [b]
gro; Bcl-7like	Df(1)9A4-5/P{EPgy2}BCL7-like^{EY10009}; gro^{1}/gro^{C105}	16.4 ± 0.35	(30) [b]
Ac3; *CG8924*	Df(1)sd72b/P{EPgy2}CG8924^{EY00245}; P{EPgy2}Ac3^{EY10141}/Df(2L)Exel7081	18.0 ± 0.33	(20) [a]
Ac3; *CG6297*	P{EPgy2}Ac3^{EY10141}/Df(2L)Exel7081; P{SUPor-P}JIL-1^{KG02848} /P{SUPor-P}JIL-1^{KG08229}	14.5 ± 0.3	(24) [b]
Ac3; *CG1943*	P{EPgy2}Ac3^{EY10141}/Df(2L)Exel7081; P{EPgy2}CG1943^{EY04914}	10.5 ± 0.33	(20) [b]
Ac3; *CG8436*	P{EPgy2}Ac3^{EY10141}/Df(2L)Exel7081; Df(3R)BSC24/P{EPgy2}EY05475	17.0 ± 0.5	(15) [a]
Ac3; Bcl7-like	P{EPgy2}BCL7-like^{EY10009}; P{EPgy2}Ac3^{EY10141}	9.0 ± 0.41	(22) [b]
Bcl7-like; *CG12299*	P{EPgy2}BCL7-like^{EY10009}; P{EPgy2}CG12299^{EY01579}/ Df (2L)Exel7820	9.5 ± 0.25	(20) [b]
Bcl7-like; *CG1832*	P{EPgy2}BCL7-like^{EY10009}; P{SUPor-P}CG1832^{KG00473}	16.7 ± 0.42	(21) [a]

Letter a indicates no statistically significant difference among mutants with an overgrowth phenotype and b a significantly reduction of double mutants compared to single mutants ($p \leq 0.01$).

Gene name, chromosomal location, function
Allelic combination — Bouton number (type 1b, muscle 13)
Control (Deficiency/+) — mean ± SE (n)

Supplementary Table S2: Synaptic growth in mutants of genes differentially regulated in ewg^{l1} mutants

Control (y w)		12.7 ± 0.3	(20)

Down-regulated genes in ewg^{l1} mutants

CG2207, Df31	39E, chromatin remodeling (Decondensation Factor 31)		
$Df31^{k05815}/Df(2L)Exel7081$		11.45 ± 0.5	(20)
Df(2L)Exel7081/+		11.4 ± 0.38	(25)
CG11307	78E1, glycosyl transferase		
$PBac\{WH\}CG11307^{f05271}/PBac\{WH\}CG11307^{f05271}$		10.1 ± 0.36	(21)
$PBac\{WH\}CG11307^{f05271}/Df(3L)ED4978$		12. ± 0.32	(21)
Df(3L)ED4978/+		11.5 ± 0.45	(20)
CG7581, Bub3	99B7, mititoc checkpoint, neuroblast progression		
$PBac\{WH\}CAP\text{-}D2^{f03381}/PBac\{WH\}CAP\text{-}D2^{f03381}$		embryonic lethal	
$PBac\{WH\}CAP\text{-}D2^{f03381}/Df(3R)01215$		embryonic lethal	
CG12299	32A3, transcription factor		
$P\{EPgy2\}CG12299^{EY01579}/P\{EPgy2\}CG12299^{EY01579}$		16.24 ± 0.22	(21)
$Df(2L)Exel7820/P\{EPgy2\}CG12299^{EY01579}$		15.0 ± 0.24	(23)[a]
Df(2L)Exel7820/+		13.13 ± 0.26	(20)
CG4916, me31B	31B1, RNA helicase		
$P\{lacW\}me31B^{k06607}/P\{lacW\}me31B^{k06607}$		20.2 ±0.53	(21)*
$Df(2L)Exel6026/P\{lacW\}me31B^{k06607}$		9.4 ± 0.25	(23)[a]
Df(2L)Exel6026/+		12.5 ± 0.35	(20)
CG5460, hairless	92F3, transcriptional co-repressor		
$H^{1}/P\{EPgy2\}H^{EY03696}$		9.1 ± 0.32	(23)[a]
$H^{25}/P\{EPgy2\}H^{EY03696}$		7.9 ± 0.38	(10)[a]
H^{1}/H^{25}		8.6 ± 0.22	(6)[a]
$P\{EPgy2\}H^{EY03696}/P\{EPgy2\}H^{EY03696}$		8.70 ±27	(23)[a]
CG8383, Pinin	85E9, alternative splicing factor		
P{EPgy2}EY06979/P{EPgy2}EY06979		7.3 ± 0.18	(20)

Gene name, chromosomal location, function<br>Allelic combination<br>Control (Deficiency/+)		Bouton number (type 1b, muscle 13)<br>mean ± SE	(n)
Df(3R)by62/P{EPgy2}EY06979		7.2 ± 0.32	(20)[a]
Df(3R)by62/+		11.1 ± 0.29	(22)
CG7471, Rpd3	64B12, histone H3 deacetylase		
P{PZ}Rpd3^{04556} */P{PZ}Rpd3*^{04556}		8.2 ± 0.29	(22)
Df(3L)Exel7208/P{PZ}Rpd3^{04556}		8.5 ± 0.32	(21)[a]
Df(3L)Exel7208/+		11.71 ± 0.21	(21)
CG9241, Mcm10	39B1, chromatin remodeling		
P{SUPor-P}Mcm10^{KG00233}*/P{SUPor-P}Mcm10*^{KG00233}		embryonic lethal	
Df(2L)Exel6047/P{SUPor-P}Mcm10^{KG00233}		9.6 ± 0.42	(22)[a]
Df(2L)Exel6047/+		11.2 ± 0.25	(20)
CG1515	7C3, vesicle membrane fusion		
P{lacW}l(1)G0155^{G0155}*/P{lacW}l(1)G0155*^{G0155}		embryonic lethal	
Df(1)ct4b1/P{lacW}l(1)G0155^{G0155}*; TM6*		9.2 ± 0.40	(20)[a]
P{lacW}l(1)G0155^{G0155}*/+; TM6*		13.25 ± 0.24	(20)
Df(1)ct4b1/+		13.35 ± 0.2	(20)
CG3429, swallow	5F4, RNP component		
swa^{3}		18.1 ± 0.47	(17)*
swa^{3} */Df(1)JF5*		9.5 ±1.3	(20)[a]
Df(1)JF5/+		12.1 ± 0.19	(20)
CG7646	76D8, neurocalcin		
P{GT1}CG7646^{BG02179}*/Df(3L)ED4858*		8.4 ± 0.64	(20)[a]
Df(3L)ED4858/+		12.95 ± 0.39	(21)
CG1943	84B2		
P{EPgy2}CG1943^{EY04914}*/P{EPgy2}CG1943*^{EY04914}		18.2 ± 0.26	(24)
Df(3R)1833/P{EPgy2}CG1943^{EY04914}		12.4 ± 0.26	(20)
Df(3R)1833/+		10.5 ± 0.3	(20)
CG1506, Ac3	39E, Adenylyl cyclase		
P{EPgy2}Ac3^{EY10141}*/Df(2L)Exel7081*		17.35 ± 0.57	(20)[a]
P{EPgy2}EY03838/Df(2L)Exel7081		17.06 ± 0.53	(15)[a]
P{EPgy2}Ac3^{EY10141}*/P{EPgy2}EY03838*		17.7 ± 0.34	(20)[a]

Gene name, chromosomal location, function<br>Allelic combination<br>Control (Deficiency/+)		Bouton number (type 1b, muscle 13)<br>mean ± SE	 (n)
Df(2L)Exel7081/+		11.4 ± 0.38	(25)
CG8384, groucho	96F10, transcriptional co-repressor		
gro^{1}/*gro*^{1}		20.0 ± 0.35	(21)[a]
gro^{1}/*gro*^{C105}		16.6 ± 0.19	(21)[a]
CG8924	13F14, transcription factor		
P{EPgy2}CG8924^{EY00245}/*P{EPgy2}CG8924*^{EY00245}		13.6 ± 0.37	(23)
Df(1)sd72b/P{EPgy2}CG8924^{EY00245}		18.7 ± 0.37	(21)[a]
Df(1)sd72b/+		13.0 ± 0.2	(20)
CG12372, spt4	49B10, chromatin remodeling, transcription elongation		
P{lacW}spt4^{k05316}/*P{lacW}spt4*^{k05316}		embryonic lethal	
Df(2R)Exel7121) /P{lacW}spt4^{k05316}		embryonic lethal	
CG4771	94C4, RNA binding		
PBac{WH}HP1c^{f04929}/*PBac{WH}HP1c*^{f04929}		7.9 ± 0.22	(14)
Df(3R)Exel6192/PBac{WH}HP1c^{f04929}		8.9 ± 0.24	(20)[a]
Df(3R)Exel6192/+		13.0 ± 0.18	(21)
CG11228, hippo	56D13, kinase		
hippo^{JM1}/*hippo*^{BF33}		die at early 2nd instar	
CG3431, Uch-L3	67B9, ubiquitin C-terminal hydrolase		
P{lacW}Uch-L3^{j2B8}/*P{lacW}Uch-L3*^{j2B8}		8.5 ± 0.46	(9)
Df(3L)Ac1/P{lacW}Uch-L3^{j2B8}		10.8 ± 0.3	(19)[b]
Df(3L)Ac1/+		12.1 ± 0.27	(23)
CG5581, otefin	55C1, inner nuclear membrane component		
PBac{5HPw^{+}*}Ote*^{B279}/*PBac{5HPw*$^{+}$*}Ote*^{B279}		11.24 ± 0.46	(25)
Df(2R)PC29/PBac{5HPw^{+}*}Ote*^{B279}		9.5 ± 0.3	(22)[a]
Df(2R)PC29/+		12 ± 0.243	(20)
CG13440, rig	57A8, ecdysone regulated transcription		
P{lacW}rig^{k07917}/*P{SUPor-P}smi21F*^{KG01076}		8.9 ± 0.22	(22)[a]
CG10901, oskar	85B7, RNP component		

Gene name, chromosomal location, function<br>Allelic combination<br>Control (Deficiency/+)		Bouton number (type 1b, muscle 13)<br>mean ± SE	(n)
osk$^{1/}$*osk*^{6}		15 ± 0.48	(20)[a]
CG1832	40C2, transcription factor		
P{SUPor-P}CG1832^{KG00473}*/P{SUPor-P}CG1832*^{KG00473}		17.1 ± 0.34	(22)
Df(2L)C1/P{SUPor-P}CG1832^{KG00473}		15.04 ± 0.29	(21)[a]
Df(2L)C1/+		12.4 ± 0.22	(20)
CG6759, cdc16	94E9, ubiquitin protein ligase		
P{EPgy2}EY12544/P{EPgy2}EY12544		10.9 ± 0.27	(22)*
Df(3R)Exel6280/P{EPgy2}EY12544		15.15± 0.34	(20)[a]
Df(3R)Exel6280/+		13.55± 0.26	(20)
CG12109, Caf1-180	7F8, Chromatin Assembly Factor 1		
P{GT1}BG02681/P{GT1}BG02681		7.9 ± 0.2	(20)
Df(1) KA14/P{GT1}BG02681		8.2 ± 0.25	(22)[a]
Df(1) KA14/+		13.3 ± 0.8	(15)
CG14217	18D1-2, Ser/Thr kinase (JNK signaling)		
P{EP}Tao-1^{EP1455}*/P{EP}Tao-1*^{EP1455}		15.1 ± 0.24	(20)*
Df(1)JA27/P{EP}Tao-1^{EP1455}		9.85 ± 0.32	(20)[a]
Df(1)JA27/+		13.3 ± 0.57	(22)
CG8436	85D5		
P{EPgy2}EY05475/P{EPgy2}EY05475		16 ± 0.28	(21)
Df(3R)BSC24/P{EPgy2}EY05475		19 ± 0.38	(21)[a]
Df(3R)BSC24/+		15.45 ± 0.23	(20)
CG2926	83B3, ring finger protein, nucleic acid binding		
PBac{RB}CG2926^{e01499}*/PBac{RB}CG2926*^{e01499}		10.7 ± 0.32	(21)
Df(3R)Exel6144/PBac{RB}CG2926^{e01499}		10.2 ± 0.29	(21)[a]
Df(3R)Exel6144/+		15.65 ±0.28	(20)
CG5940, CycA	68E1, G2-M transition		
P{PZ}CycA^{03946}*/P{PZ}CycA*^{03946}		embryonic lethal	
Df(3L)Exel6115/P{PZ}CycA^{03946}		embryonic lethal	
CG6297, Jil1	68A5, histone H3 kinase		

Gene name, chromosomal location, function<br>Allelic combination<br>Control (Deficiency/+)		Bouton number (type 1b, muscle 13)<br>mean ± SE	(n)
P{SUPor-P}JIL-1^{KG08229}*/P{SUPor-P}JIL-1*^{Scim}		15.1 ±0.35	(21)[a]
P{SUPor-P}JIL-1^{KG02848}*/P{SUPor-P}JIL-1*^{KG08229}		16.3± 0.3	(22)[a]
P{SUPor-P}JIL-1^{Scim}*/P{SUPor-P}JIL-1*^{KG02848}		16.0 ± 0.41	(21)[a]
Df(3L)vin2/P{SUPor-P}JIL-1^{KG02848}		15.1 ± 0.32	(24)[a]
CG4184, Arc105	21D1, PolII tyranscription mediator		
PBac{WH}MED15^{f04180}*/PBac{WH}MED15*^{f04180}		embryonic lethal	
Df(2L)S2/PBac{WH}MED15^{f04180}		7.7 ± 0.33	(21)[a]
Df(2L)S2/+		12.2 ±0.36	(21)
CG8589	50F1, RNA binding		
P{EPgy2}EY08611/P{EPgy2}EY08611		15.0 ± 0.45	(27)
Df(2R)Exel7131/17479		7.8 ± 0.25	(20)[a]
Df(2R)Exel7131/+		11.45 ± 0.31	(20)
CG9999, Sd	37E1, segregation distorter, nucleocytoplasmic transport		
P{EP}RanGap^{EP1173}*/P{EP}RanGap*^{EP1173}		8.55 ± 0.29	(20)
Df(2L)Exel8041/P{EP}RanGap^{EP1173}		8.0 ± 0.2	(20)[a]
Df(2L)Exel8041/+		13.5 ± 0.27	(20)
CG2446	10D7		
P{EPgy2}CG2446^{EY00672}*/P{EPgy2}CG2446*^{EY00672}		16.41 ± 0.32	(24)
Df(1)Exel6242/P{EPgy2}CG2446^{EY00672}		14.3 ± 0.26	(22)
Df(1)Exel6242/+		13.5 ± 0.27	(20)
CG5807	96A23, lipocalin, lipid binding		
P{EPgy2}CG5807^{EY06164}*/P{EPgy2}CG5807*^{EY06164}		13.4 ± 0.26	(21)
Df(3R)Exel6100/P{EPgy2}CG5807^{EY06164}		8.4 ± 0.36	(22)[a]
Df(3R)Exel6100/+		11.3 ± 0.33	(20)
CG6875, asp	96A19, abnormal spindle, microtubule bound kinase		
P{EPgy2}EY07687P{EPgy2}EY07687		8.9 ± 0.34	(22)
Df(3R)96B/P{EPgy2}EY07687		8.4 ± 0.42	(20)[a]
Df(3R)96B/+		12.1 ± 0.13	(20)
CG5300, Klp31E	31E1		
P{EPgy2}EY08009/P{EPgy2}EY08009		17.2 ± 0.4	(24)*

Gene name, chromosomal location, function<br>Allelic combination<br>Control (Deficiency/+)		Bouton number (type 1b, muscle 13)<br>mean ± SE	 (n)
Df(2L)J106/P{EPgy2}EY08009		10.6 ±0.32	(22)[a]
Df(2L)J106 /+		12.4 ± 0.29	(20)
CG12750	36E2, RNA binding		
P{GT1}CG12750^{BG01636}*P{GT1}CG12750*^{BG01636}		11.4 ± 0.35	(20)
Df(2L)Exel8038/P{GT1}CG12750^{BG01636}		10.7 ± 0.35	(22)[c]
Df(2L)Exel8038/+		11.45 ± 0.31	(22)
CG15835	43F1, transcription co-factor		
P{SUPor-P}CG15835^{KG04636}*P{SUPor-P}CG15835*^{KG04636}		13.9 ± 0.24	(20)
Df(2R)Exel6055/P{SUPor-P}CG15835^{KG04636}		14.2 ± 0.25	(20)
CG13599	95C13		
P{EPgy2}CG13599^{EY08519}*/P{EPgy2}CG13599*^{EY08519}		11.8 ± 0.32	(20)
Df(3R)Exel6196/P{EPgy2}CG13599^{EY08519}		8.1 ±0.22	(20)[c]
Df(3R)Exel6196/+		9.6 ± 0.25	(20)
Bcl7-like, CG17252	8D12		
P{EPgy2}BCL7-like^{EY10009}*/P{EPgy2}BCL7-like*^{EY10009}		20.55 ± 1	(20)
Df(1)9A4-5/P{EPgy2}BCL7-like^{EY10009}		18.6 ± 0.44	(20)[a]
Df(1)9A4-5/+		12.8 ± 0.24	(20)
CG6311	74D2		
P{SUPor-P}CG6311^{KG01392}*/P{SUPor-P}CG6311*^{KG01392}		17.1 ± 0.29	(21)
Df(3L)Exel6132/P{SUPor-P}CG6311^{KG01392}		13.2 ± 0.26	(20)
Df(3L)Exel6132/+		12.8 ± 0.22	(20)
Rox8, CG5422	95D9, RNA binding		
P{EPgy2}Rox8^{EY04187}*/P{EPgy2}Rox8*^{EY04187}		13.3 ± 0.29	(21)
Df(3R)Exel6197/P{EPgy2}Rox8^{EY04187}		7.95 ± 0.27	(20)[a]
Df(3R)Exel6197/+		10.48 ± 0.32	(21)

Up-regulated genes in *ewg*^{l1} mutants

CG2803	60E8, troponin C, akin-1		
P{SUPor-P}Tina-1^{KG05573}*/P{SUPor-P}Tina-1*^{KG05573}		10.1 ± 0.4	(22)
Df(2R)M60E/P{SUPor-P}Tina-1^{KG05573}		14.25 ± 0.27	(20)

Gene name, chromosomal location, function<br>Allelic combination<br>Control (Deficiency/+)		Bouton number (type 1b, muscle 13)<br>mean ± SE	 (n)
Df(2R)M60E/+		13.0 ± 0.25	(22)
CG6746	33B6, protein phosphatase		
P{EP}CG6746^{EP1118}*/P{EP}CG6746*^{EP1118}		12.4 ± 0.26	(20)
Df(2L)Exel6031/P{EP}CG6746^{EP1118}		14.7 ± 0.3	(21)
Df(2L)Exel6031/+		13.3 ±0.45	(21)
CG11312, inscutable	57B3, cytoskeletal adaptor protein		
insc^{P49}*/P{PZ}insc*^{05475}		embryonic lethal	
insc^{P49}*/P{lacW}insc*^{k12405}		embryonic lethal	
P{lacW}insc^{k12405}*/P{PZ}insc*^{05475}		embryonic lethal	
CG8213	45D7, trypsin-like protease		
P{EPgy2}ced-6^{EY11592}*/P{EPgy2}ced-6*^{EY11592}		10.4 ± 0.27	(20)
Df(2R)11/P{EPgy2}ced-6^{EY11592}		13.1 ± 0.27	(20)
Df(2R)11/+		12.5 ± 0.26	(22)
CG4306	75C6		
P{EPgy2}EY06480/P{EPgy2}EY06480		19.8 ± 0.34	(23)
Df(3L) ED225/P{EPgy2}EY06480		13.7 ± 0.24	(21)
Df(3L)Cat/P{EPgy2}EY06480		13.7 ± 0.3	(21)
Df(3L)ED225/+		13.2 ± 0.2	(20)
Df(3L)Cat/+		10.5 ± 0.5	(21)
CG6724	32A5, WD repeat adapter protein		
PBac{RB}CG6724^{e02149}*/Df(2L)Exel7820*		embryonic lethal	
P{EPgy2}CG6724^{EY15313}*/P{EPgy2}CG6724*^{EY15313}		8.0 ±0.31	(24)
PBac{RB}CG6724^{e02149}*/P{EPgy2}CG6724*^{EY15313}		7.5± 0.36	(21)[a]
CG7399, Henna	66A12, phenylalanine/tyrosine hydoxylase,		
Hn^{r3}		6.6 ± 0.26	(21)
Hn^{r3} */Hn*^{r}		8.3 ± 0.39	(22)[a]
Hn^{r3}*/Df(3L)pbl-X1*		9.0 ± 0.41	(24)[a]
Df(3L)pbl-X1/+		13.0 ± 0.22	(21)
CG7230, ribbon	56C6, transcription factor		
rib^{1}*/rib*^{2}		embryonic lethal	

Gene name, chromosomal location, function<br>Allelic combination<br>Control (Deficiency/+)		Bouton number (type 1b, muscle 13)<br>mean ± SE	(n)
CG1463	11B13		
P{EPgy2}EY00581/P{EPgy2}EY00581		11.9 ± 0.23	(20)
Df(1)JA26/P{EPgy2}EY00581		14.1 ±0.27	(22)
Df(1)JA26/+		12.9 ± 0.3	(20)
CG5575, ken	60A6, transcription factor		
P{PZ}ken^{1}/P{PZ}ken^{02970}		10.6 ± 0.34	(20)
Df(2R)106/P{PZ}ken^{02970}		embryonic lethal	
Df(2R)106/P{PZ}ken^{1}		8.5 ± 0.3	(21)[a]
Df(2R)106/+		15.2 ± 0.35	(20)
CG9847, Fkbp13	57E3, prolyl cis-trans isomerase		
P{PZ}Fkbp13^{00734}/P{PZ}Fkbp13^{00734}		embryonic lethal	
Df(2R)Exel6076/ P{PZ}Fkbp13^{00734}		8.6 ±0.19	(21)[a]
Df(2R)Exel6076/+		13.1 ± 0.25	(20)
CG18321, miple2	61B2, Dmidkine2		
P{EPgy2}EY04126/P{EPgy2}EY04126		20.8 ± 1	(20)
Df(3L)emc-E12/P{EPgy2}EY04126		8.5 ± 0.35	(13)[a]
Df(3L)emc-E12/+		10.7 ± 0.37	(20)
Df(3L)Exel6084/P{EPgy2}EY04126		9.2 ± 0.25	(21)[a]
Df(3L)Exel6084/+		12.0 ± 0.25	(21)
CG3800	59B6, transcription factor		
P{SUPor-P}KG05669/P{SUPor-P}KG05669		10.6 ± 0.34	(22)
Df(2R)WI213/P{SUPor-P}KG05669		11.2 ± 0.4	(21)[b]
Df(2R)WI213/+		14.1 ± 0.27	(20)
CG4445	42E7, pGant3, GalNAc transferase3		
P{SUPor-P}pgant3^{KG01548}/P{SUPor-P}pgant3^{KG01548}		12.8 ± 0.38	(22)
Df(2R)Drl-rv3/P{SUPor-P}pgant3^{KG01548}		9.9 ± 0.3	(20)[a]
Df(2R)Drl-rv3/+		15.6 ± 0.3	(20)
CG10077	65D3, RNA helicase		
PBac{PB}CG10077^{c04490}/PBac{PB}CG10077^{c04490}		13.5 ± 0.28	(20)

Gene name, chromosomal location, function<br>Allelic combination<br>Control (Deficiency/+)		Bouton number (type 1b, muscle 13)<br>mean ± SE	(n)
Df(3L) W4.5/PBac{PB}CG10077^{c04490}		16.0 ± 0.34	(24)[b]
Df(3L) W4.5/+		14.6 ±0.34	(21)
CG4758	30F5, SRP-dependent membrane targeting		
P{lacW}l(2)k13305^{k13305}*/P{lacW}l(2)k13305*^{k13305}		embryonic lethal	
Df(2L)BSC50/P{lacW}l(2)k13305^{k13305}		10.25 ± 0.35	(15)[a]
Df(2L)BSC50/+		12.7 ± 0.21	(20)
CG8896, 18wheeler	56F8, cell adhesion		
18w$^{\Delta 7\text{-}35}$*/P{lacW}18w*^{k02701}		16.77 ± 0.538	(22)[a]
CG7708	91B8, Na-proline symporter		
P{EPgy2}EY07667/Df(3R)Exel6180		12.0 ± 0.28	(21)
CG32701 (CG2994)	8E7, ER directed protein synthesis		
P{lacW}l(1)G0320^{G0320}*/P{lacW}l(1)G0320*^{G0320}		embryonic lethal	
P{SUPor-P}l(1)G0320^{KG03129}*/P{SUPor-P}l(1)G0320*^{KG03129}		7.35 ± 0.3	(20)[a]
P{lacW}l(1)G0320^{G0320}*/P{SUPor-P}l(1)G0320*^{KG03129}		9 ± 0.29	(14)
CG8434, lambik	52F2, cell adhesion		
P{EPgy2}EY11328/P{EPgy2}EY11328		11.2 ± 0.34	(21)
Df(2R)Jp6/P{EPgy2}EY11328		19.7 ± 0.58	(22)[a]
Df(2R)Jp6/+		13.35 ± 0.2	(20)
CG7896	99D3, cell adhesion		
P{GT1}CG7896^{BG00791}*/P{GT1}CG7896*^{BG00791}		8.8 ± 0.25	(22)
Df(3R)X3F/P{GT1}CG7896^{BG00791}		11.2 ± 0.4	(20)[b]
Df(3R)X3F/+		13.4 ± 0.26	(20)
CG3903 Gliotactin	35D4, cell adheshion, septate junctions		
P{EPgy2}Gli^{EY11600}*/P{EPgy2}Gli*^{EY11600}		14.5 ± 0.32	(20)
P{EPgy2}Gli^{EY11600}*/Gli*^{1}		19.05 ± 0.25	(20)[a]
Gli^{1}*/Df(2L)Sco*^{rv14}		embryonic lethal	
CG18485, stumps	88C8, scaffolding protein involved in FGF signaling		
P{PZ}09904a, ***stumps***^{09904b}*/P{PZ}09904a,* ***stumps***^{09904b}		embryonic lethal	
Df(3R)red1/P{PZ}09904a, ***stumps***^{09904b}		13.3 ± 0.38	(20)

Gene name, chromosomal location, function<br>Allelic combination<br>Control (Deficiency/+)		Bouton number (type 1b, muscle 13)<br>mean ± SE	(n)
Df(3R)red1/P{PZ}09904a, $stumps^{09904b}$		13.25± 0.38	(20)
CG4115	87B7-8, cell adhesion		
PBac{PB}$CG4115^{c05379}$*/PBac{PB}*$CG4115^{c05379}$		9.1 ± 0.26	(21)
Df(3R)Exel6164/PBac{PB}$CG4115^{c05379}$		11.5 ± 0.36	(21)[b]
Df(3R)Exel6164+/		14.6 ± 0.24	(21)
CG11212	42A13, patched related receptor		
P{XP}d00738/P{XP}d00738		5.8 ±0.26	(23)
PBac{RB}Ptr^{e02155}*/P{XP}d00738*		5.6 ± 0.3	(22)[a]
P{XP}d00738/Df(2R)ED1552		7.0 ± 0.39	(24)[a]
Df(2R)ED1552/PBac{RB}Ptr^{e02155}		embryonic lethal	
Df(2R)ED1552/+		15.1 ± 0.32	(21)

[a, b, c] Letters indicate statistically significant differences (a≤0.0001, b≤0.001 and c≤0.05) of transheterozygous mutants (chromosomal deficiency or second allele) compared with controls (y w and heterozygotes for chromosomal deficiency). As a control, chromosomal deficiencies were tested transheterozygous with y w. Since they remove several genes they could affect synaptic growth as dosage effect in a combinatorial manner due to clustering of functionally related genes. Alleles kept as balanced stocks due to lethality or sterility were not tested as transheterozygotes with y w as they unlikely accumulate genetic modifiers and also origin from different genetic backgrounds.
Genes where transheterozygous mutant combinations showed additional phenotypes were: pupal lethality: CG7471, CG6724, CG9847, CG4758, CG8896 and CG3431, morphological aberrations: CG5460, CG8384, CG12109, CG17252, CG7399, CG5575 and CG13440, sterility: CG3429, CG10901, CG6297, CG5300, CG32701 and CG6875, flightlessness: CG4916 and CG7646.

* As these alleles are viable and kept as homozygous stocks, they most likely have accumulated genetic modifiers that are lost upon analysis as transheterozygotes for a chromosomal deficiency.

Standard genetic markers (y, w, ry), which have no effect on synaptic growth, are not shown.

Supplemental Table S3: Fold Change of ewg compared to wildtype (CS) and rescue (elav EWG, NS) arrays

probe set	gene	Accession	ewg/CS fold change	ewg/elavEWG fold change
152816_at	CG2207 (7D5-7D5; 1491;)	FBgn0029990	1.58	1.95
152252_at	CG5282 (77B9-77C1; 1512;)	FBgn0036986	1.81	1.73
153689_at	CG11307 (78E5-78E5; 1420;)	FBgn0037106	1.5	1.52
150128_at	CG14312 (90F7-90F7; 327;)	FBgn0038596	1.95	1.89
154168_at	Bub3 (99B9-99B9; 1113;)	FBgn0025457	1.51	1.43
143212_at	l(1)10Bb (10B8-10B8; 914; lethal (1) 1Bb)	FBgn0001491	1.82	1.66
155152_at	CG12299 (32A3-32A3; 3309;)	FBgn0032295	1.5	1.65
143692_at	Dhh1 (67D12-67D12; 3398; DEAD/DEAH RNA helicase 1)	FBgn0011802	1.51	2.11
150974_at	CG2381 (102A6-102A6; 904;)	FBgn0039900	3.16	3.31
153626_at	BcDNA:LD08534 (32D1-32D1; 801;)	FBgn0027890	1.64	1.72
147355_at	CG17290 (54A2-54A2; 327;)	FBgn0034201	1.5	1.163
151688_s_at	LD3221.	FBgn0001169_EST	1.52	1.65
149351_at	CG2051 (83C1-83C1; 1850;)	FBgn0037376	1.5	1.55
153983_at	CG4880 (15B1-15B1; 1688;)	FBgn0030803	1.53	1.62
151461_r_at	CG13303 (102B8-102B8; 120;)	FBgn0041005	1.58	1.38
149576_at	CG8383 (85E6-85E6; 912;)	FBgn0037737	1.54	1.61
146018_at	CG7851 (29A4-29A4; 1391;)	FBgn0032013	1.94	2.09
146018_at	CG7851 (29A4-29A4; 1391;)	FBgn0032013	1.92	2.15
154479_at	CG8507 (85E9-85E9; 1363;)	FBgn0037756	1.52	1.63
147514_at	CG11797 (56E4-56E4; 531;)	FBgn0034468	2.4	2.65
153325_at	Rpd3 (64B17-64B17; 2141;)	FBgn0015805	1.5	1.59
150975_at	CG10322 (102A7-102A8; 630;)	FBgn0039901	2.21	2.48
155096_at	CG17149 (77A4-77A4; 3558;)	FBgn0036955	1.42	1.57
146648_at	CG8245 (41E3-41E3; 1294;)	FBgn0033031	2.57	3.15
155063_at	CG9241 (39B4-39B4; 2793;)	FBgn0032929	1.65	1.9
146159_at	CG5337 (31E2-31E2; 2782;)	FBgn0032249	1.51	1.67
153571_at	XRCC1 (4F2-4F2; 2283;)	FBgn0026751	1.33	1.7
143692_at	8241 (67D12-67D12; 3398; DEAD/DEAH RNA helicase 1)	FBgn0011802	1.57	2.11
145019_at	CG32365 (12A4-12A4; 994;)	FBgn0030501	1.62	1.5
154675_at	CG1515 (7C8-7C8; 1299;)	FBgn0029978	1.52	1.72
148373_at	CG7504 (66B10-66B10; 4254;)	FBgn0035842	1.5	1.66
151660_at	LD21771	FBgn0003655_EST	1.51	1.65
149084_at	CG7646 (76E2-76E2; 832;)	FBgn0036926	3.29	4.08
149420_at	CG1943 (84C1-84C1; 937;)	FBgn0037468	1.5	1.62
142749_at	Ac3 (39E3-39E3; 5046;)	FBgn0023416	3.85	4.81
142039_s_at	LD33829	FBgn0001139_EST	1.55	1.72
141219_at	CG7099 (34B6-34B6; 5958;)	FBgn0032517	1.51	1.77
153942_at	CG8924 (13F14-13F14; 2155;)	FBgn0030710	1.53	1.64
142904_at	spt4 (49B5-49B5; 944;)	FBgn0028683	1.52	1.69
154828_at	CG4771 (94C6-94C6; 2320;)	FBgn0039018	1.53	1.65
154828_at	CG4771 (94C6-94C6; 2320;)	FBgn0039018	1.51	1.71
154255_at	Uch-L3 (67B5-67B5; 1137; Ubiquitin C-terminal hydrolase)	FBgn0011327	1.55	1.78
154965_at	Ote (55C3-55C4; 1554; Otefin)	FBgn0003022	1.53	1.79
153804_at	CG11881 (99A1-99A1; 2116;)	FBgn0039638	1.54	1.77
142991_at	CG13440 (57B2-57B2; 1977;)	FBgn0034531	1.52	1.84
142612_at	CG11760 (85B2-85B2; 503;)	FBgn0037615	1.56	1.74
154312_at	osk (85C1-85C1; 2247; oskar)	FBgn0003015	1.52	1.82
146613_at	CG1832 (40C2-40C2; 2068;)	FBgn0032979	1.5	1.61
154801_at	CG8290 (48D7-48D8; 5670;)	FBgn0033670	1.54	1.76
153927_at	CG8460 (28F1-28F1; 1307;)	FBgn0031996	1.355	1.74
153457_at	CG4570 (86C7-86C7; 1274;)	FBgn0037844	1.51	1.79
144093_at	cdc16 (94E11-94E11; 2585;)	FBgn0025781	1.54	1.74
143730_at	Gbp (55C13-55C13; 1775; GTP-binding-protein)	FBgn0013969	1.5	1.72
141513_at	CG12109 (7F4-7F5; 3647;)	FBgn0030054	1.5	1.72
153995_at	CG14217 (18D5-18D6; 4030;)	FBgn0031030	1.52	1.72
154835_at	CG10110 (51A5-51A6; 4574;)	FBgn0033941	1.358	1.74
145017_at	CG1573 (12A2-12A2; 1234;)	FBgn0030497	1.351	1.76
154612_at	CG8436 (85D4-85D4; 940;)	FBgn0037670	1.56	1.78
153821_at	CG16975 (34A8-34A9; 3952;)	FBgn0032475	1.44	1.76

probe set	gene	Accession	ewg/CS fold change	ewg/elavEWG fold change
152154_at	CG7227 (28D11-28D11; 2110;)	FBgn0031970	-1.5	-1.55
154780_at	CG6347 (50C12-50C13; 1243;)	FBgn0033874	-1.68	-1.75
154061_at	CG5171 (27F6-27F6; 1117;)	FBgn0031907	-1.71	-1.88
149382_at	CG15592 (83E2-83E2; 702;)	FBgn0037416	-1.51	-1.6
154819_at	CG2961 (9B7-9B7; 1310;)	FBgn0030187	-3.59	-3.51
146794_at	CG14756 (44B2-44B2; 711;)	FBgn0033275	-2.06	-2.07
152090_at	CG6746 (33B9-33B9; 1042;)	FBgn0032394	-1.5	-1.52
143675_at	insc (57B6-57B6; 3719; inscuteable)	FBgn0011674	-1.59	-1.61
154985_at	CG9083 (60E4-60E4; 1186;)	FBgn0035077	-5.33	-4.41
147084_at	CG13159 (49A1-49A1; 231;)	FBgn0033721	-2.39	-2.27
154231_at	CG1155 (83E2-83E2; 1065;)	FBgn0037423	-2.13	-2.08
150111_at	CG12310 (90D1-90D1; 309;)	FBgn0038562	-1.73	-1.31
149394_at	CG15189 (83E4-83E4; 801;)	FBgn0037429	-1.67	-1.59
150947_at	CG11518 (100D1-100D1; 3442;)	FBgn0039853	-1.14	-1.52
155018_at	CG8213 (45A1-45A2; 5352;)	FBgn0033359	-1.76	-1.67
148995_at	CG4306 (75C6-75C6; 989;)	FBgn0036787	-1.73	-1.66
142736_s_at	CG16796 (53A5-53B1; 1939;)	FBgn0034115	-1.72	-1.64
155122_at	CG9411 (12E8-12E8; 3656;)	FBgn0030569	-3.74	-2.87
144898_at	CG15740 (10F1-10F1; 3042;)	FBgn0030340	-3.4	-2.71
149380_at	CG1153 (83E2-83E2; 1360;)	FBgn0037414	-3.69	-2.95
153802_at	CG5864 (95D1-95D1; 1032;)	FBgn0039132	-1.5	1.58
141583_at	Cyp309a2 (51D2-51D2; 1780;)	FBgn0033980	-1.57	-2.09
152816_at	CG2233 (7D5-7D5; 1491;)	FBgn0029990	-1.58	-1.95
154890_at	CG10611 (69C2-69C2; 1507;)	FBgn0036289	-1.64	-1.3
147431_at	CG18108 (55C9-55C9; 340;)	FBgn0034329	-1.5	-1.83
153434_at	Hn (66A11-66A12; 1536; Henna)	FBgn0001208	-1.53	-1.41
155018_at	CG8869 (45A1-45A2; 5352;)	FBgn0033359	-1.76	-1.67
149988_at	CG5470 (89B1-89B1; 257;)	FBgn0038384	-1.66	-1.91
151802_at	Adh (35B3-35B3; 1504; Alcohol dehydrogenase)	FBgn0000055	-1.5	-1.69
154821_at	CG8147 (85D21-85D21; 1846;)	FBgn0037699	-1.51	-1.91
149663_at	CG6633 (86D4-86D4; 1680;)	FBgn0037861	-1.35	-1.53
146084_at	CG3752 (30B4-30B4; 1997;)	FBgn0032114	-1.31	-1.67
143988_at	Lip1 (32A1-32A1; 1563; Lipase 1)	FBgn0023496	-1.75	-1.37
141583_at	Cyp6a20 (51D2-51D2; 1780;)	FBgn0033980	-1.62	-2.09
151795_at	CG7176 (66C8-66C8; 2033;)	FBgn0035881	-1.36	-1.55
151643_at	LD1658.	FBgn0034424_EST	-1.63	-1.4
148268_at	CG13297 (65A5-65A5; 414;)	FBgn0035685	-2.14	-1.66
154049_at	CG9370 (70F4-70F4; 2494;)	FBgn0036444	-1.57	-1.36
154981_at	CG10211 (37A1-37A1; 3691;)	FBgn0032685	-1.55	-1.34
147333_at	CG15615 (53D8-53D8; 1101;)	FBgn0034159	-2.67	-1.83
148393_at	CG7076 (66C8-66C8; 489;)	FBgn0035875	-1.78	-1.45
143299_at	Pepck (55E1-55E1; 2481; Phosphoenolpyruvate carboxykinase)	FBgn0003067	-1.93	-1.52
153953_at	CG1463 (82A3-82A3; 1218;)	FBgn0037224	-3.01	-2.03
141386_at	ImpL1 (70A5-70A5; 1149; Ecdysone-inducible gene L1)	FBgn0001256	-2.08	-1.58
151996_at	CG33009 (59B4-59B4; 4470;)	FBgn0034797	-1.68	1.08
152407_at	CG9390 (43F8-43F9; 7450;)	FBgn0033245	-1.67	-1.19
152923_at	CG10585 (78A6-78A6; 1955;)	FBgn0037044	-1.97	-1.52
150933_at	CG3684 (100B9-100C1; 1293;)	FBgn0039834	-1.63	-1.37
152222_at	BcDNA:GH09147 (42E2-42E2; 2859;)	FBgn0027558	-2.61	-1.5
145670_at	CG3131 (23B2-23B2; 4764;)	FBgn0031464	-1.62	-1.34
152444_at	ken (60A6-60A7; 3056; ken and barbie)	FBgn0011236	-2.46	-1.64
148416_at	CG6458 (66D10-66D10; 781;)	FBgn0035919	-1.71	-1.3
148302_at	CG8638 (65E4-65E4; 507;)	FBgn0035736	-1.83	-1.61
144455_at	CG15239 (4A1-4A1; 1482;)	FBgn0029681	-2.03	-1.74
150676_at	CG5480 (97C2-97C2; 852;)	FBgn0039446	-2.25	-1.8
150177_at	CG6026 (91E4-91E4; 4573;)	FBgn0038676	-1.57	-1.34
149395_at	CG15188 (83E4-83E4; 843;)	FBgn0037430	-2.95	-2.14
148235_at	CG10546 (64F1-64F1; 975;)	FBgn0035636	-1.86	-1.6
141384_at	CG12164 (43A4-43A4; 1383;)	FBgn0033158	-1.81	-1.56

154649_at	CG11305 (98F10-98F10; 2990;)	FBgn0039631	1.52	1.68	154666_at	CG32548 (23F3-23F3; 2426;)	FBgn0031540	-1.56	-1.31
141256_at	mus210 (51F4-51F5; 4281; mutagen-sensitive 21)	FBgn0004698	1.59	1.81	150970_at	CG1629 (102A3-102A3; 1158;)	FBgn0039896	-1.53	-1.39
142566_at	CG2926 (83B4-83B4; 7382;)	FBgn0037344	1.5	1.75	152208_at	Fkbp13 (57E6-57E6; 927;)	FBgn0010470	-1.79	-1.56
154702_at	CG4973 (92C4-92C4; 1373;)	FBgn0038772	1.52	1.77	143606_at	Cyp18a1 (17D1-17D1; 2730; Cytochrome P45-18a1)	FBgn0010383	-2.14	-1.77
141233_at	CG5966 (5C10-5D1; 2596;)	FBgn0029831	1.66	1.4	149389_at	CG1157 (83E2-83E3; 948;)	FBgn0037424	-5.27	-2.67
155068_at	CycA (68D4-68D4; 1833; Cyclin A)	FBgn0000404	1.54	1.8	148235_at	CG10546 (64F1-64F1; 975;)	FBgn0035636	-1.86	-1.6
141583_at	Cyp6a20 (51D2-51D2; 1780;)	FBgn0033980	1.62	2.09	153033_at	CG7675 (91A2-91A2; 1100;)	FBgn0038610	-1.53	-1.36
151702_r_at	LD387.3	FBgn0030969_EST	1.5	1.9	148152_at	CG15005 (64B2-64B2; 2280;)	FBgn0035508	-1.5	-1.3
151702_r_at	LD387.3	FBgn0030969_EST	1.5	1.9	153953_at	CG14639 (82A3-82A3; 1218;)	FBgn0037224	-3.01	-2.03
153444_at	JIL-1 (68A3-68A3; 5643;)	FBgn0020412	1.55	1.79	154454_at	CG8867 (23C1-23C1; 5202;)	FBgn0031481	-1.5	-1.31
152313_at	Cyp6a17 (51D2-51D2; 1519;)	FBgn0015714	1.5	1.69	153951_at	ImpE2 (63E1-63E1; 1630; Ecdysone-inducible gene E2)	FBgn0001254	-1.68	-1.45
153854_at	CG4184 (85E8-85E8; 1790;)	FBgn0037748	1.5	1.72	153408_at	CG14557 (97A1-97A1; 1174;)	FBgn0039414	-1.82	-1.55
147406_at	CG10910 (55B1-55B1; 1407;)	FBgn0034289	2.51	3.51	148417_at	CG6469 (66D10-66D10; 423;)	FBgn0035920	-1.72	-1.49
147193_at	CG8589 (50F2-50F3; 1680;)	FBgn0033921	1.55	1.79	152137_at	CG9747 (99E3-99E3; 2124;)	FBgn0039754	-2.02	-1.35
153879_at	Sd (37E3-37E3; 2089; Segregation distorter)	FBgn0003346	1.5	1.7	147919_at	CG18321 (61B2-61B2; 1117;)	FBgn0035123	-1.75	-1.39
151869_at	CG2446 (10D4-10D5; 2447;)	FBgn0030328	1.53	1.73	148726_at	CG13737 (70C5-70C5; 237;)	FBgn0036382	-1.52	-1.3
145084_at	CG9521 (13A1-13A1; 1866;)	FBgn0030588	1.52	1.74	142508_at	CG3800 (59B6-59B6; 1327;)	FBgn0034802	-1.59	-1.32
154448_at	C1159686C5-86C5; 1511;)	FBgn0037834	1.5	1.65	152222_at	BcDNA:GH09147 (42E2-42E2; 2859;)	FBgn0027558	-2.75	-1.42
154866_at	asp (96A23-96A23; 6284; abnormal spindle)	FBgn0000140	1.5	1.7	151712_at	LD43683	FBgn0031097_EST	-2.49	-1.48
153726_at	CG3878 (18D3-18D3; 1728;)	FBgn0031025	1.58	1.77	144088_at	scf (61F5-61F5; 1579; supercoiling factor)	FBgn0025682	-1.55	-1.38
151610_at	LD3548.	FBgn0029547_EST	1.5	1.68	143663_at	Trp1 (30F5-30F5; 2050; Translocation protein 1)	FBgn0011584	-1.83	-1.33
142157_at	CG7014 (88D8-88D8; 847;)	FBgn0038277	1.5	1.76	152907_at	18w (56F9-56F9; 5422; 18 wheeler)	FBgn0004364	-1.74	-1.32
141602_at	CG12750 (36F7-36F7; 3887;)	FBgn0032678	1.53	1.97	151892_at	CG7708 (91B8-91B8; 2357;)	FBgn0038641	-1.8	-1.33
142811_at	CG15835 (43F5-43F5; 1537;)	FBgn0033233	1.51	1.67	153163_at	Roe1 (50B6-50B6; 1042;)	FBgn0014877	-1.54	-1.24
151898_at	karyopherin-alpha1 (76D2-76D3; 2210; karyopherin alpha1)	FBgn0024889	1.53	1.72	154368_at	CG2994 (8E7-8E8; 1229;)	FBgn0030135	-2.09	-1.41
155002_at	CG110926C4-96C5; 8238;)	FBgn0039302	1.51	1.75	148178_at	CG15022 (64B12-64B12; 828;)	FBgn0035547	-2.6	-1.53
152045_at	Cyp4c3 (100B1-100B2; 1932; Cytochrome P45-4c3)	FBgn0015032	1.54	1.72	143988_at	Lip1 (32A1-32A1; 1563; Lipase 1)	FBgn0023496	-1.75	-1.37
141310_at	CG9682 (99E5-99E5; 1449;)	FBgn0039760	1.53	1.73	142796_at	CG8306 (53C7-53C8; 1937;)	FBgn0034142	-1.82	-1.4
154822_at	CG3797 (75F4-75F4; 2766;)	FBgn0036842	1.56	1.79	153986_at	CG8434 (52F5-52F5; 4186;)	FBgn0034083	-1.53	-1.28
142122_at	CG1168 (83A5-83A5; 942;)	FBgn0037318	1.56	1.31	149468_at	CG2781 (84E5-84E5; 831;)	FBgn0037534	-2.56	-1.64
151634_at	LD12333	FBgn0024921_EST	1.51	1.29	154520_at	CG7896 (99D3-99D3; 4294;)	FBgn0039728	-1.9	-1.41
150472_at	CG13599 (95C12-95C12; 888;)	FBgn0039128	1.55	1.29	143221_at	Gli (35D6-35D6; 3820; Gliotactin)	FBgn0001987	-1.5	-1.26
153451_at	Cdk5 (52A13-52A14; 1586; Cyclin-dependent kinase 5)	FBgn0013762	1.54	1.68	142813_at	stumps (88C9-88C9; 3727)	FBgn0020299	-1.86	-1.4
154079_at	BCL7-like (8D12-8D12; 1653;)	FBgn0026149	1.56	1.35	147903_at	CG9358 (60E7-60E7; 366;)	FBgn0035089	-1.58	-1.29
151237_at	CG13230 (47D4-47D4; 219;)	FBgn0040764	1.76	1.46	148541_at	CG14147 (68A1-68A1; 468;)	FBgn0036112	-2	-1.45
153949_at	CG2608 (38E1-38E1; 1440;)	FBgn0032870	1.51	1.42	149752_at	CG4115 (87B11-87B11; 982;)	FBgn0038017	-1.84	-1.37
148962_at	CG6311 (74D2-74D2; 2487;)	FBgn0036735	1.54	1.46	150930_at	CG15560 (100B8-100B8; 1982;)	FBgn0039826	-1.5	-1.4
154361_at	CG8352 (65B4-65B5; 1728;)	FBgn0035705	1.58	1.31	154015_at	CG4060 (89F2-89F2; 1107;)	FBgn0038487	-2.55	-1.56
152871_at	CG1909 (102C5-102C5; 2183;)	FBgn0039911	2.53	2.04	147042_at	CG8986 (48C7-48C7; 616;)	FBgn0033658	-2.22	-1.48
141733_at	CG4118 (73A7-73A7; 2757;)	FBgn0036640	1.53	1.71	154890_at	CG10657 (69C2-69C2; 1507;)	FBgn0036289	-1.64	-1.3
145019_at	CG11177 (12A4-12A4; 994;)	FBgn0030501	1.62	1.34	152675_at	CG1681 (11F1-11F1; 1098;)	FBgn0030484	-1.82	-1.36
143568_at	Rox8 (95D5-95D5; 3165; Rox8)	FBgn0005649	1.57	1.32	148155_at	CG15007 (64B2-64B2; 444;)	FBgn0035511	-1.62	-1.29
143313_at	Pros29 (57B15-57B15; 956; Proteasome 29kD subunit)	FBgn0003150	1.53	1.37	146674_at	CG11212 (42A10-42A10; 3740;)	FBgn0033068	-2.27	-1.5
146034_at	CG13399 (29C3-29C3; 387;)	FBgn0032043	1.6	1.36	143298_at	CG2150 (100E3-100E3; 1146;)	FBgn0003065	-2.72	-1.6
141726_at	alien (29F8-29F8; 1477; alien)	FBgn0013746	1.54	1.35	142434_at	CG2803 (60E5-60E5; 739;)	FBgn0035083	-1.64	-1.5
149209_at	CG14565 (79A1-79A1; 810;)	FBgn0037129	1.59	1.37	141233_at	CG31522(5C10-5D1; 2596;)	FBgn0029831	-1.7	-1.41
154154_at	CG9393 (85D25-85D25; 1464;)	FBgn0037710	1.57	1.34	148152_at	CG3523(64B2-64B2; 2280;)	FBgn0035508	-1.5	-1.3
145559_at	CG4577 (21E3-21E3; 1833;)	FBgn0031306	1.64	1.54	148417_at	CG32029(66D10-66D10; 423;)	FBgn0035920	-1.72	-1.43
153802_at	CG5802 (95D1-95D1; 1032;)	FBgn0039132	1.5	1.68	146674_at	CG3132442A10-42A10; 3740;)	FBgn0033068	-1.64	-1.51
